# Design, synthesis, molecular modelling and biological evaluation of oxiconazole-based 1,2,3-triazoles as dually acting antifungal and antitumor agents

**DOI:** 10.1038/s41598-026-65234-9

**Published:** 2026-08-14

**Authors:** Azza E. Ismail, Mohamed I. Ashmawy, Michael G. Shehat, Mustafa A. Al-Seqely, Mona H. Badr, Sherif A. F. Rostom

**Affiliations:** 1 Pharmaceutical Chemistry Department, Faculty of Pharmacy, Alexandria University, Alexandria, 21521 Egypt; 2 Microbiology and Immunology Department, Faculty of Pharmacy, Alexandria University, Alexandria, 21521 Egypt

**Keywords:** Drug repurposing, Oxiconazole-based 1,2,3-triazoles, Dual-acting antifungal–anticancer agents, Azole resistance, *CYP51* inhibition, Biochemistry, Biotechnology, Cancer, Chemical biology, Chemistry, Drug discovery, Microbiology

## Abstract

The rise of azole-resistant and the elevated prevalence of fungal infections in immuno-compromised cancer patients emphasize the necessity for strategies that can tackle both fungal resistance and tumor expansion. We postulated that the appropriate alteration of the azole pharmacophore through the inclusion of a 1,2,3-triazole ring might result in dual-acting drugs that preserve antifungal efficacy while exhibiting anticancer properties. In accordance with the aforementioned reasoning, a targeted library of oxiconazole-derived 1,2,3-triazoles was synthesized and assessed for antifungal potency, cytotoxicity, and mechanism of action. Different analogues (**4**, **5b, 6, 8, 10, 11, 18,** and **19c**) exhibited significant antifungal activity against fluconazole-sensitive C. *albicans* strains (S1, S2), with minimum inhibitory concentrations (MICs) ranging from 16 to 64 μg/mL, while reduced activity was observed against resistant strains (S3, S4). Compound **11**, the nearest structural counterpart of oxiconazole, proved to be the most powerful alternative, markedly suppressing ergosterol biosynthesis in both susceptible (S1) and resistant (S3) strains by 65.65% and 61.89%, respectively. Checkerboard assays demonstrated additive to indifferent relationships between **11** and fluconazole, with fractional inhibitory concentration index (FICI) values of 2 and 0.75 against S1 and S3, respectively. Docking study revealed that **11** occupied the lanosterol 14α-demethylase active pocket. In addition, compounds (**5b, 5c, 7–9, 11, 13, 16, 17**, and **19c**) had moderate to modest cytotoxic activity against A549, Caco-2, and MCF7 cancer cells, with IC_50_ values of 33.66 to 100 µM in comparison to docetaxel. Compounds (**7, 8,** and **11**) were particularly active, with compound **11** triggering S-phase cell-cycle arrest and apoptosis in A549 cells. **11**, a chemo sensitizing adjuvant, significantly lowered the IC_50_ of docetaxel in A549 and Caco-2 cells by 87.98% and 43.35%, respectively. Combination index (CI) analysis revealed strong synergism (CI = 0.16) in A549 cells and moderate synergism (CI = 0.58) in Caco-2 cells at the 50% effect level (Fa = 0.5), confirming that the combination is more effective than either agent alone. Overall, these data indicate compound **11** as a mechanistically validated dual antifungal-anticancer lead that bridges azole optimization and adjuvant oncology methods, necessitating further structure–activity optimization and in vivo testing.

**Supplementary Information:**

The online version contains supplementary material available at 10.1038/s41598-026-65234-9.

## Introduction

Invasive fungal infections (IFIs) have been rapidly growing globally, with documented terrifying mortality rates, reaching 20 –70% in immunocompromised patients^1,2^. In this context, patients under anticancer chemotherapy are significantly immunocompromised and are highly susceptible to opportunistic fungal infections^3^. Meanwhile, virulence factors and toxins associated with untreated chronic fungal infections can initiate significant damage to host cells, subsequent prolonged inflammatory response and impairment of immunity, that would end with the development of cancerous tumors^4,5^.

Different classes of antifungal agents are clinically available in the global market including polyenes, echinocandins, pyrimidines, and allylamines. However, the clinical application of some members of these groups has been limited due to their undesirable side effects and the development of resistant fungal strains^6–8^. Currently, synthetic azoles including imidazoles (e.g. oxiconazole (**I**)), and triazoles (e.g. fluconazole), have stemmed as the most widely used antifungal agents, because of their significant wide spectrum, cost-effectiveness, and acceptable safety profile^9,10^. Suppressing lanosterol 14α-demethylase (LDM, CYP51), the azole’s primary target, causes fungal membrane damage, inhibiting cell growth and accumulating toxic lanosterol^11,12^. Azoles bind to LDM through two moieties: (a) the metal binding group (MBG), represented by the imidazole N^3^ and triazole N^4^, which coordinate with the heme iron, and (b) the scaffold counterpart that occupies the enzyme’s binding site^11^.

In recent years, 1,2,3-triazoles have emerged as essential structural motifs in medicinal chemistry, with outstanding metabolic stability, structural rigidity, and varied hydrogen-bonding capabilities^13,14^. These attributes have been successfully utilized in the development of antifungal agents^15–17^ as well as anticancer *candida*tes with antiproliferative, cytotoxic, and kinase-inhibitory actions, against multidrug-resistant cancer cell lines^18–23^.

It has been well-documented that co-administration of several medications by individuals suffering from cancer associated with fungal infections, may lead to additional health complications, especially in those with compromised liver and/or kidney functions. These problems could be overcome by utilizing compound combination, which have long been recognized to disrupt erroneous behavior in some biological systems^24,25^ e.g. blending antifungals and antibiotics to address serious infections^26^, beside some complicated network-driven conditions e.g. cancer^27^. Therefore, the concept of monotherapy with a single drug that performs dual actions could be advantageous from both clinical and financial perspectives^28^. Such dual-acting drugs may offer advantages in overcoming resistance, simplifying treatment regimens, and improving patient compliance^24^. While these strategies have yielded substantial results, few have directly addressed the clinical burden of fungal infections and cancer-related complications. Recently, there is a growing proof suggesting that certain azole antifungals inhibit cancer cell proliferation, presumably due to shared molecular vulnerabilities between fungal sterol biosynthesis and cancer-associated signaling pathways^29–31^. This convergence provides a compelling argument for developing single molecular entities that have both antifungal and anticancer activities.

**Fig. 1:**
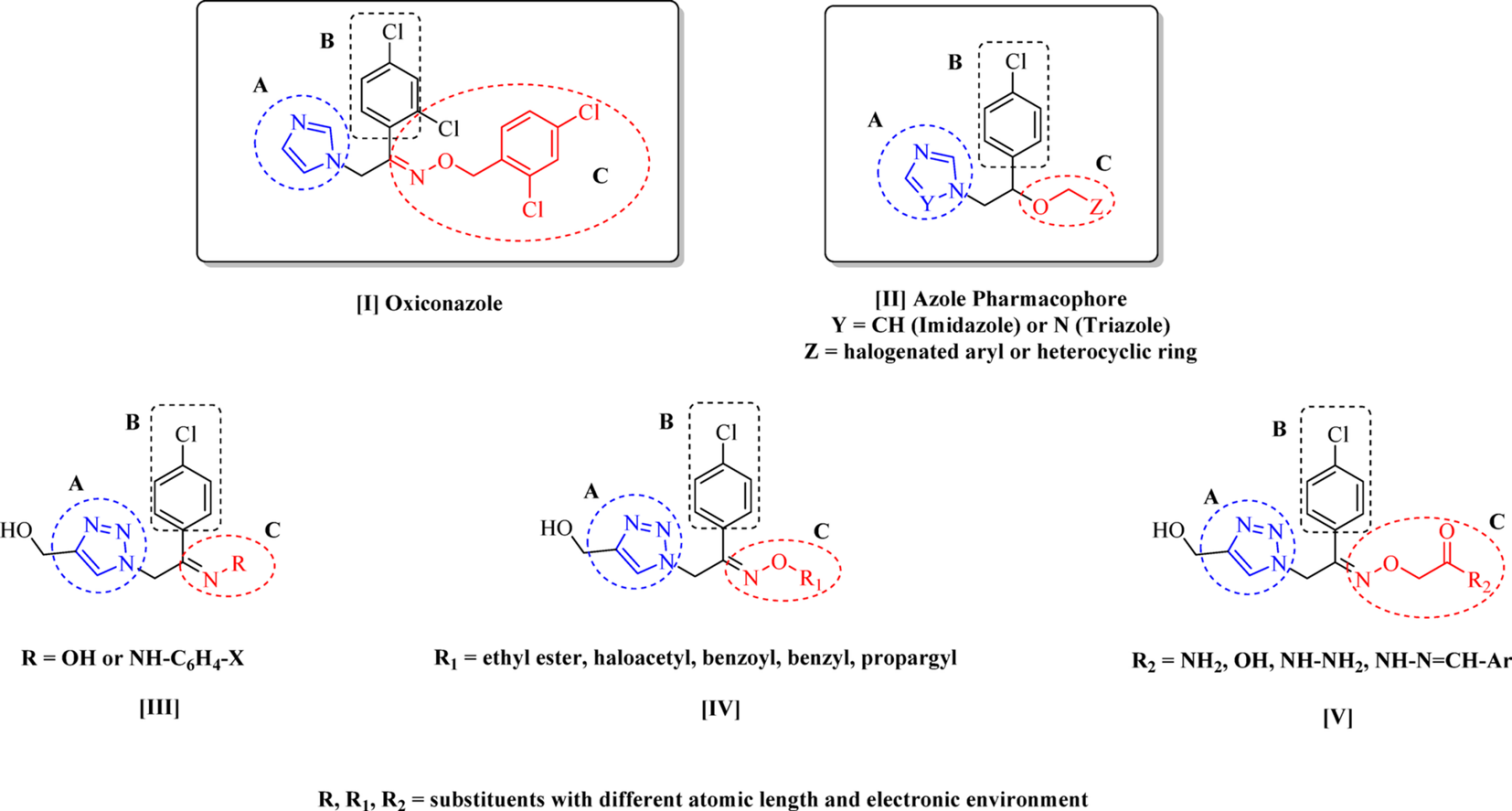
[**I**] Oxiconazole, [**II**] the azole pharmacophore, [**III-V**] general structures of the target compounds.

In view of the above-mentioned facts, and in continuation of our efforts in the development of novel chemotherapeutic agents^28,32–38^, we report herein the design and synthesis of some novel 1,2,3-triazole derivatives belonging to the general structures (**III-V)** (Fig. 1) to be evaluated for their antifungal and cytotoxic potentials. The target compounds (**III-V)** were tailored to involve the basic structure of oxiconazole (**I**) (Fig. 1)^39^, where the imidazole ring was replaced by a hydroxymethyl-1,2,3-triazole moiety (**A**) that would bind heme iron via triazole-*N*^*3*^, while keeping the 4-chlorophenyl ring (**B**) and oxyimino functionality untouched. Additionally, the tail counterpart (**C**) was intended to comprise various aliphatic and/or aromatic moieties linked to the phenethyl azole substructure through different linkers and spacers. Such functionalities represent different electronic, lipophilic, and steric environments, aiming to interact with an additional pocket in the fungal LDM enzyme’s active site^40^, and would specifically address the resistance-related constraints of conventional azoles.

The in vitro antifungal activity of all the newly synthesized compounds was evaluated employing the microbroth dilution Eucast method^41^, and the ergosterol quantification assay^42^ to assess the possible mechanism of antifungal action. Furthermore, the effect of combining the most active antifungal member with fluconazole was also studied. On the other hand, the cytotoxic potential of the same twenty target compounds was investigated in a panel of three cancerous cell lines namely, lung cancer (A549), colon cancer (CaCo-2), and breast cancer (MCF-7) utilizing the MTT assay^43^. Moreover, the influence of the most active compound on the progress of the cell cycle and apoptosis induction in lung cancer (A549) was examined in a trial to explore the plausible mechanism behind its antiproliferative potential. Additionally, the possible synergistic effect of combining the most active compound with docetaxel was also investigated. Finally, a molecular docking study was conducted to the most active compound to determine its likely binding affinity to the *Candida* 14α-demethylase enzyme (PDB code: 5TZ1) from a molecular standpoint. In addition, *in-silico* computation studies were carried out to assess compounds’ suitability to serve as possible orally active drug candidates.

## Result and discussion

### Chemistry

**Scheme 1:**
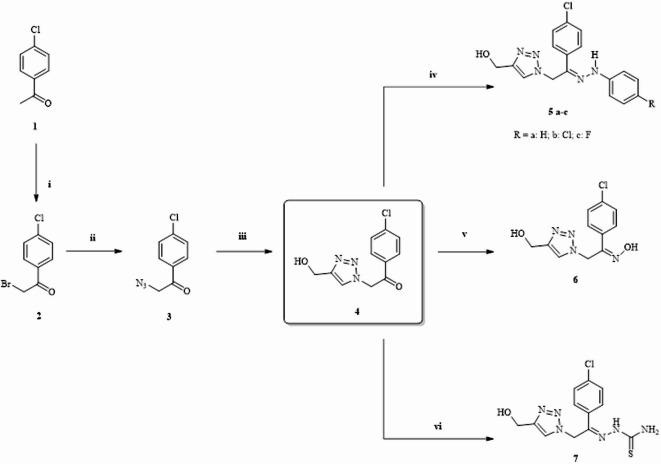
Reagents and reaction conditions: **i**: NBS, lactic acid, 100 °C, **ii**: NaN_3_, acetone/H_2_O, RT, 3 h;** iii**: Propargyl alcohol, THF/H_2_O, CuSO_4_.5H_2_O, Na ascorbate, RT, 1 h;** iv**: Pheny; hydrazine derivatives, piperidine, ethanol, reflux, 8 h;** v**: NH_2_OH.HCl, Na acetate, ethanol, reflux, 3 h;** vi**: Thiosemicarbazide, piperidine, ethanol, reflux 7 h.

**Scheme 2:**
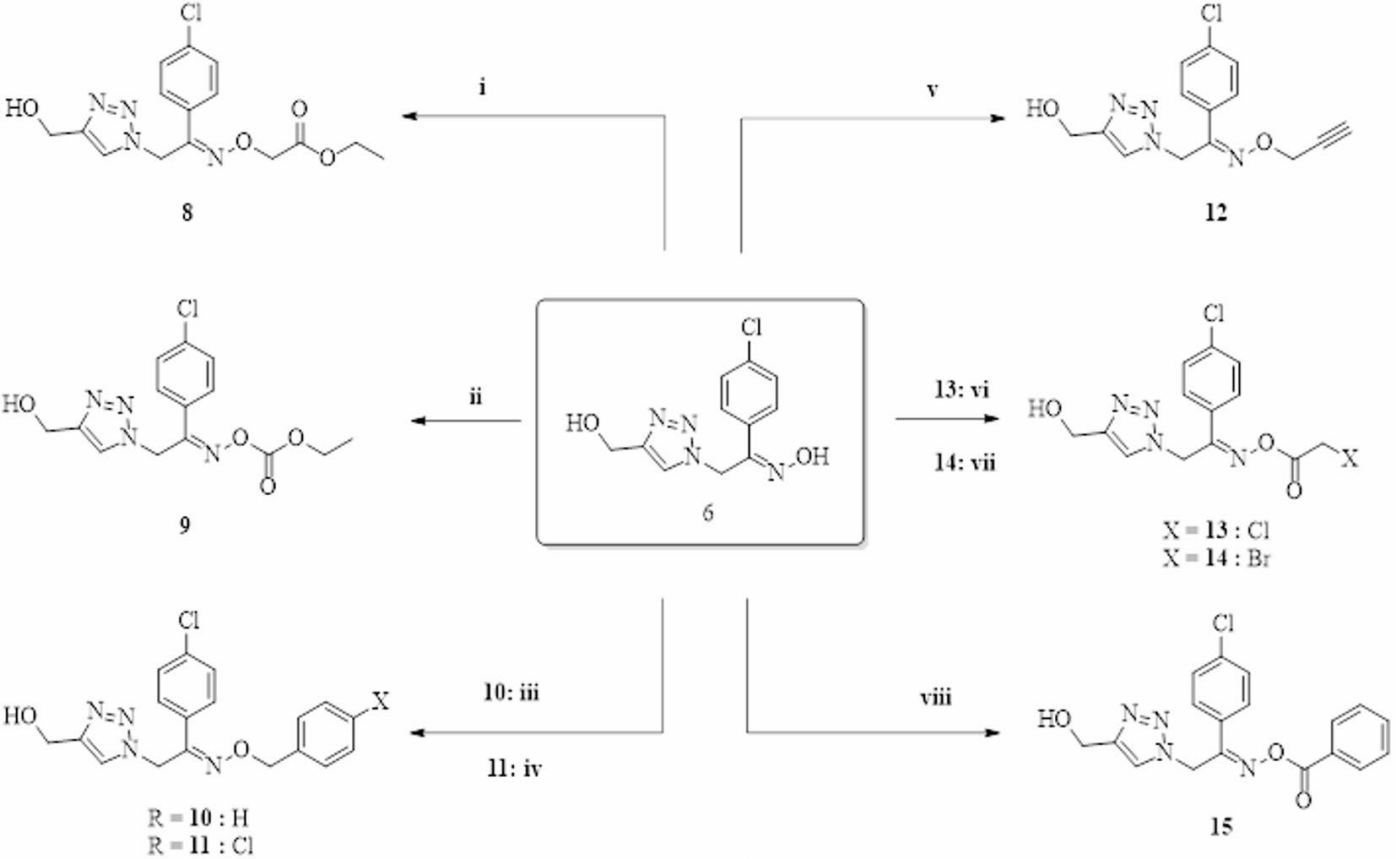
Reagents and reaction conditions:** i**: Ethyl bromoacetate, K_2_CO_3_, acetonitrile, RT, 24 h;** ii**: Ethyl chloroformate, K_2_CO_3_, KI, acetonitrile, RT, 2 h;** iii**: Benzyl bromide, K_2_CO_3_, acetonitrile, RT, 12 h;** iv**: 4-Chlorobenzyl chloride, K_2_CO_3_, KI, acetone, RT, 24 h;** v**: Propargyl bromide, KOH, DMSO, 60 °C, 4 h;** vi**: Chloroacetyl chloride, K_2_CO_3_, KI, acetone, RT, 12 h;** vii**: Bromoacetyl bromide, K_2_CO_3_, acetone, RT, 12 h;** vii**: Benzoyl chloride, K_2_CO_3_, KI, acetone, RT, 6 h.

**Scheme 3:**
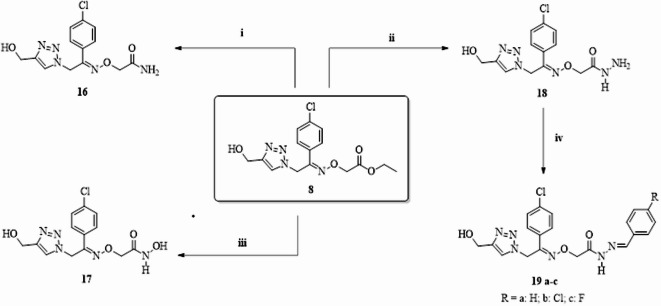
Reagents and reaction conditions:** i**: Conc NH_4_OH, ethanol, RT, 48 h;** ii**: NH_2_NH_2_.H_2_O, ethanol, RT, 3 h;** iii**: NH2OH.HCl, KOH, ethanol, RT, 24 h; vi: Benzaldehyde derivatives, ethanol, reflux, 3 h.

The synthetic strategies employed to obtain the intermediate and target compounds are illustrated in Schemes 1, 2, 3. In Scheme 1, the key intermediate ketone **4** was synthesized using three-step procedure. Initially, 4-chloroacetophenone **1** was α-brominated in lactic acid at 100 °C using N-bromo succinimide (NBS) to yield the corresponding 4-chlorophenacyl bromide **2**^44^. The latter **2** was then reacted with sodium azide in acetone at room temperature to give the corresponding 4-chlorophenacyl azide **3**, which when treated with propargyl alcohol in THF using copper sulfate and sodium ascorbate as catalysts yielded the requested intermediate 1,4-disubstituted 1,2,3-triazoles **4**^45^. Condensing **4** with various Phenylhydrazine derivatives in ethanol using piperidine as a basic catalyst furnished the corresponding hydrazones **5a-c**. Moreover, the oxime **6** was achieved by refluxing **4** with hydroxylamine hydrochloride in ethanol in the presence of sodium acetate^46^. Furthermore, the thiosemicarbazone derivative **7** was obtained by reacting **4** with thiosemicarbazide in alkaline medium using piperidine. It is worth mentioning that the thiosemicarbazone **7** displayed two doublets at 7.86 and 7.61 ppm assigned for NH_2_ non-equivalent protons. A logical explanation for this could be that the intramolecular hydrogen bonding of one of the N-4 protons and the imine nitrogen, resulting in a pair of distinct doublets for the NH_2_ protons^47^.

In Scheme 2, the oxime **6** was further reacted with various alkylating agents namely; ethyl bromoacetate, ethyl chloroformate, benzyl bromide, 4-chlorobenzyl chloride, chloroacetyl chloride, bromoacetyl bromide, or benzoyl chloride in acetone in the presence of anhydrous potassium carbonate to obtain the targeted compounds **8–11**, **13–15;** respectively^48^. Analogously, reacting **6** with propargyl bromide in DMSO in the presence of potassium hydroxide afforded the propargyl derivative **12**.

Shifting to Scheme 3, conversion of the ester **8** into different carboxylic acid derivatives was achieved via reaction with concentrated ammonia, hydroxylamine and potassium hydroxide or hydrazine hydrate in ethanol to obtain the targeted amide **16,** hydroxamic acid **17** and acid hydrazide **18;** respectively^48^. Finally, refluxing **18** with the appropriate benzaldehyde derivative in ethanol resulted in the formation of the corresponding Schiff bases **19a-c**^33^.

### Biological evaluation

#### In vitro antifungal activity against *Candida albicans* strains

All the newly synthesized compounds were evaluated for their in vitro antifungal activity against four *C. albicans* strains including three clinical isolates namely, sensitive *Candida* strain (S1), moderate resistant *Candida* strain (S2), fluconazole resistant *Candida* strain (S3) and one standard fluconazole resistant *Candida* strain (ATCC 10231) (S4)^49^. The conventional microbroth dilution Eucast method was utilized to calculate the minimum inhibitory concentration (MIC) which denotes the first well exhibiting a 50% drop in growth relative to the drug-free well’s growth. Compounds with MIC > 128 mg/mL were considered inactive against any of the tested four *Candida* strains. Fluconazole was employed as a reference antifungal drug and dimethyl sulfoxide (DMSO) was used as a solvent.

The results presented in Table 1 revealed that all the investigated compounds displayed variable inhibitory effects on the growth of the tested four *C. albicans* strains. In general, most of the tested compounds exhibited a remarkably better antifungal profile against S1, S2 and S3 fungal strains as compared to the resistant S4 strain. In particular, the S1 strain showed relative high sensitivity towards the tested compounds. In this respect, sixteen of the tested analogs displayed MIC value of 16 μg/mL counting for 50% of the activity of fluconazole (MIC 8 μg/mL) against the S1 strain, whereas the remaining four compounds **5a**, **7**, **15** and **19a** showed mild or even no activity (MIC ≥ 128 μg/mL). Concerning the S2 strain, thirteen compounds (**4**, **5b**, **5c**, **6**, **8**, **9**, **10**, **11**, **14**, **17**, **18**, **19b** and **19c**) were able to demonstrate a distinctive growth inhibitory potential (MIC 16 μg/mL), being fourfold more potent than fluconazole (MIC 64 μg/mL). Meanwhile, the analogs **12** and **16** (MIC 32 μg/mL) were twofold more potent than the reference drug, whereas the remaining five compounds were mildly active or inactive against the S2 strain (MIC ≥ 128 μg/mL). Regarding the activity against the resistant *Candida* strain (S3), except compounds **5a**, **13** and **19a,** all the tested seventeen analogs exhibited distinctive to moderate activities (MIC range 16—128 μg/mL) when compared with fluconazole which was completely inactive (MIC > 128 μg/mL). Finally, the resistant strain (S4) showed the least sensitivity towards the investigated compounds, when compared with the other three *Candida* strains. In detail, compound **11** stemmed as the most active member against this strain with MIC 32 μg/mL. Meanwhile, eleven compounds (**4**, **5b**, **6**–**8**, **10, 12**, **16**, **18**, **19b** and **19c**) showed moderate antifungal potential (MIC 64 μg/mL), whereas the rest eight compounds showed weak, or no activity (MIC ≥ 128 μg/mL), nevertheless remain better than fluconazole (MIC > 128 μg/mL) against this strain.

**Table 1:** Minimal inhibitory concentrations (MIC, μg/mL) of the newly synthesized compounds against *C. albicans* strains.

Compound no	S^a^	S2^b^	S3^c^	S4^d^
4	16	16	16	64
5a	> 128^e^	> 128	> 128	> 128
5b	16	16	16	64
5c	16	16	16	128
6	16	16	16	64
7	128	128	128	64
8	16	16	16	64
9	16	16	16	> 128
10	16	16	16	64
11	16	16	16	32
12	16	32	16	64
13	16	> 128	> 128	> 128
14	16	16	16	> 128
15	128	128	128	> 128
16	16	32	32	64
17	16	16	16	> 128
18	16	16	16	64
19a	> 128	> 128	> 128	> 128
19b	16	16	32	64
19c	16	16	16	64
FLC^f^	8	64	> 128	> 128

^a^S1: Sensitive *Candida* strain, ^b^S2: Moderate resistant *Candida* strain, ^c^S3: Fluconazole resistant *Candida* strain, ^d^S4 Standard fluconazole resistant *Candida* strain (ATCC 10,231), ^e^Inactive: MIC > 128 μg/mL, ^f^FLC: Fluconazole.

The obtained antifungal activity of the tested compounds could be correlated to the adopted structural variations and modifications. The key precursor ethanone **4** showed significant antifungal activity against the tested four *C. albicans* strains. Replacement of this carbonyl with different functionalities proved to be essential in modulating the antifungal activity of the targeted compounds. In this respect, conversion of the carbonyl function in **4** into the corresponding phenylhydrazone (**5a;** R = H) led to a complete abolishment of the activity. Whereas introducing a halogen atom at position-4 of the phenyl ring (**5b**; R = Cl and **5c**; R = F) resulted in a significant enhancement of the antifungal potential. Furthermore, the activity of the precursor **4** was kept almost unchanged upon introducing an oxime functionality as in the analog **6**. However, derivatization of the ketone **4** into the thiosemicarbazone **7** led to an obvious reduction in the activity against all *Candida* strains except S4. Further modification in the oxyimino derivative **6** by introducing an ester moiety as in the analogs **8** and **9** (ethyl acetate and ethyl formate, respectively), did not offer any significant advantage to the targeted antifungal activity. Moreover, alkylating **6** with benzyl, 4-chlorobenzyl or bromoacetyl groups (compounds **10**, **11** and **14**; respectively) kept the activity at same level. However, introducing propargyl, chloroacetyl or benzoyl moieties (compounds **12**, **13** and **15**; respectively) resulted in an obvious reduction in the antifungal potential. Taking the ethyl acetate derivative **8** as another key intermediate, upon treatment with different amines (compounds **16**–**18**) the targeted antifungal activity was variably affected. In this respect, the amide **16** showed a twofold decrease in the activity against S2 and S3 fungal strains. Whereas, the corresponding hydroxamic acid derivative **17** retained the same potential against S1-S3 strains, together with a complete loss of activity against the S4 strain. Meanwhile, the acid hydrazide analog **18** possessed the same antifungal profile as the parent compound **8**. Further derivatization of compound **18** into the corresponding Schiff bases furnished three analogs **19a-c** with inconstant antifungal potential having the following order of activity: **19c** (R = F) > **19b** (R = Cl) > **19a** (R = H).

#### Ergosterol inhibition assay

The most active antifungal compound **11** was further investigated for its in vitro ability to inhibit ergosterol biosynthesis in the fungal cellular membrane (the characteristic mechanism of action of azole antifungal agents). Two strains were utilized: the sensitive strain (S1) and the fluconazole-resistant strain (S3), where fluconazole was used as a positive control. This test relies on the quantification of the amount of ergosterol (before and after exposure to the compound) after ensuring the growth of *Candida*. The percentage reduction in ergosterol content was calculated according to reported equations^42,50^ and the results are represented in Table 2.

**Table 2:** Inhibition of ergosterol biosynthesis in *candida* isolates (^a)^S1 and ^b)^S3) by compound **11** and fluconazole.

	Test compound	Mean ergosterol content	% Reduction
S1 strain	Negative control	0.02044	–
	Compound **11** MIC (16 µg/ml)	0.00702	65.65
	Compound **11** 0.5 MIC (8 µg/ml)	0.01671	18.26
	^c^ FLC MIC (8 µg/ml)	0.00106	94.78
	FLC 0.5 MIC (4 µg/ml)	0.01071	47.61
S3 strain	Negative control	0.03666	–
	Compound **11** MIC (16 µg/ml)	0.01397	61.89
	Compound **11** 0.5 MIC (8 µg/ml)	0.01999	45.46
	FLC MIC (256 µg/ml)	0.00303	91.73
	FLC 0.5MIC (128 µg/ml)	0.03074	16.12

^a)^S1: Sensitive *Candida* strain, ^b)^S3: Fluconazole resistant *Candida* strain, ^c)^FLC: Fluconazole,

In general, the results obtained demonstrated that ergosterol inhibition was dose-dependent in both sensitive as well as resistant *Candida* isolates, as previously reported with several natural and synthetic substances^51,52^. Concerning the S1 strain, a significant reduction in ergosterol content (65.65%) was exhibited by compound **11** at its MIC, while at 0.5 MIC, it showed only 18.26% inhibition. On the other hand, the positive control fluconazole displayed 94.78% reduction in ergosterol content at its MIC, and nearly 50% reduction at 0.5 MIC. Regarding the S3 strain, fluconazole could produce superior reduction in the biosynthesis of ergosterol as compared to compound **11** (91% vs. 61.89%, respectively), yet the MIC of **11** was significantly lower than that of fluconazole (MIC 16 vs. 256, respectively). However, at their 0.5 MICs, the inhibitory potential of fluconazole was dramatically diminished up to 16.12%, whereas the analog **11** could keep about ¾ of its inhibitory effect (45.46% reduction) on the same strain.

#### Antifungal drug combination study

Utilizing an adjuvant to increase the responsiveness of drug-resistant *Candida* species to the antifungal activity of conventional azoles is a strategy that merits further exploration. Such combination therapy of two or more antifungal medicines, or antifungal agents with another drugs, have been tested on an experiential basis, aiming at overcoming treatment failures. The primary benefits of antifungal combinations include achieving a synergistic antifungal effect with lower dosages of each component, while simultaneously reducing harmful side effects and the emergence of fungal resistance^53^.

In this study, it was planned to investigate the impact of combining the most active antifungal *candida*te **11** with fluconazole (the standard antifungal agent) on both fluconazole-resistant and sensitive *C. albicans* clinical isolates. The conventional microdilution checkerboard assay was conducted, and the mean fractional inhibitory concentration indices (ΣFICI) were calculated^54,55^. Synergy was identified when the ΣFICI ≤ 0.5; additive effect as ΣFICI = flu 0.5–1; indifference as ΣFICI > 1–4 and antagonism when ΣFICI > 4^56^.

The results presented in Table 3 demonstrated that the combined effect of fluconazole with the analog **11** showed indifference (ΣFICI = 2) against the sensitive *Candida* strain (S1), where the MIC of both drugs were kept unchanged. Meanwhile, **11** revealed an additive effect on the activity of fluconazole against the fluconazole-resistant *Candida* strain (S3) expressing ΣFICI = 0.75, with fourfold reduction of the MIC of fluconazole. Collectively, these results negate the idea that compound **11** would be an antagonist to fluconazole.

**Table 3:** The combined antifungal effect of compound **11** and fluconazole against two *C. albicans* strains.

*C. albicans* clinical isolates	Compound 11/FLC^a^ combination		
	MIC (µg/ml)	ΣFICI^b^	Mode
S1^c^	16/8	2	IND^e^
S3^d^	8/64	0.75	ADD^f^

^a^FLC: fluconazole, ^b^ΣFICI: mean fractional inhibitory concentration index, ^c^S1: Sensitive *Candida* strain, ^d^S3: Fluconazole resistant *Candida* strain, ^e^IND: indifference, ^f^ADD: additive.

#### In vitro MTT cytotoxicity assay

The standard [3-(4,5-dimethylthiazol-2-yl)-2,5-diphenyltetrazolium bromide] (MTT) assay was employed to investigate the *in-vitro* cytotoxic effects of all the newly synthesized 20 compounds on a panel of three human cancer cell lines namely, colorectal adenocarcinoma Caco-2, human lung adenocarcinoma A549 and breast cancer MCF7 [Purchased from Nawah Scientific, Egypt (originally obtained from ATCC)]. Table 4 summarizes the findings as IC_50_ (µM), which is the concentration of the compound which inhibits the vitality of 50% of the cells within 72 h. Compounds with IC_50_ > 300 µM were considered inactive against any of the three cancer cell lines^57^.

**Table 4:** Cytotoxic effects (IC_50_; µM)^a^ ± SEM^b^ of the active compounds on three human tumor cell lines using MTT assay.

Compound no	Caco-2^c^	A549^d^	MCF7^e^
5b	62.68 ± 0.115	86.52 ± 4.585	104.8 ± 7.95
5c	> 300f.^)^	> 300	110.6 ± 6.25
7	**33.66 ± 4.97**	**48.62 ± 1.265**	**73.5 ± 1.385**
8	**40.12 ± 0.44**	**49.04 ± 0.165**	**47.14 ± 2.44**
9	71.9 ± 5.27	60.65 ± 0.545	108 ± 2.5
11	**47.82 ± 1.685**	**45.12 ± 0.495**	**47.13 ± 0.2**
13	103.5 ± 6.185	> 300	> 300
16	> 300	> 300	80.74 ± 4.965
17	54.37 ± 3.745	74.38 ± 0.51	> 300
19c	> 300	> 300	129.3 ± 1.45
DOC^g^	0.0467 ± 0.008	0.8915 ± 0.05	34.42 ± 1.005

^a^IC_50_: Concentration of the compound which inhibits the vitality of 50% of the cells within 72 h, ^b^ ± SEM: standard error of the mean ^c^ Caco-2 (Colorectal adenocarcinoma), ^d^ A549 (Human lung adenocarcinoma), ^e^ MCF7 (Breast cancer), ^f^ Totally inactive against this cell line, ^g^ DOC: docetaxel Positive control cytotoxic agent.

The obtained data revealed that ten compounds, **5b, 5c**, **7- 9**, **11**, **13, 16, 17** and **19c**, were able to affect the viability of the three tested tumor cell lines, whereas the remaining ten compounds were totally inactive. In general, the active compounds showed moderate to mild cytotoxic activities when compared to the reference drug docetaxel. Moreover, the tested tumor cell lines exhibited a variable degree of sensitivity towards the active compounds. In this respect, Caco-2 cell line showed a moderate to mild sensitivity against seven compounds namely, **5b, 7- 9**, **11**, **13** and **17**, among which the analogs **7**, **8** and **11** (IC_50_ 34.91, 40.16 and 47.81 µM, respectively) were the most active. Regarding the activity against the A549 cell line, an adequate cytotoxic potential was achieved by compounds **5b, 7- 9**, **11** and **17** (IC_50_ range 45.12—89.78 µM), where the analogs **7** and **11** (IC_50_ 45.17 and 45.12 µM, respectively), were the most convective. On the other hand, the growth of the MCF7 cell line was found to be inhibited by eight compounds namely, **5b, 5c, 7- 9**, **11**, **16** and **17** (IC_50_ range 47.31—128.3 µM) among which the analogs **8** and **11** were the most active (IC_50_ 47.76 and 47.31 µM, respectively), exhibiting nearly 67% of the activity of docetaxel (IC_50_ 31.93 µM) against this cell line. Taking both the growth inhibitory potential and the spectrum of cytotoxic activity as preference parameters, compounds **7**, **8** and **11** have stemmed as the most distinguished members in this study.

A close examination of the structures of the active compounds revealed that the phenyl hydrazone derivative **5a** was completely devoid of any cytotoxic activity. Introduction of a chlorine atom at position-4 of the phenyl ring furnished compound **5b**, with a noticeable enhancement in both cytotoxic potency and spectrum against the three tested cancer cell lines. However, isosteric replacement of the chloro substituent with a fluoro one as in the analog **5c,** led to an obvious reduction in the spectrum of activity. Furthermore, all of the three cell lines proved to be highly susceptible to the cytotoxicity of the thiosemicarbazone derivative **7**. Moreover, in spite of the inactivity shown by the oxime derivative **6,** yet the ester derivatives of the oxyimino functionality greatly improved the anticancer activity, as seen in the ethyl acetate **8** and ethyl formate derivatives **9**. When compared to the precursor **6,** introducing a benzyl moiety (**10**; R = H) showed no improvement in the activity, while the 4-chlorobenzyl analog **11** (R = Cl) exhibited a significant activity against all the tested cell lines. Additionally, incorporating propargyl, chloroacetyl, bromoacetyl or benzoyl functionalities offered compounds **12**, **13**, **14** and **15**; respectively, did not offer any advantage to the anticancer activity of the parent **6**, except the chloroacetyl analog **13** (X = Cl) which showed weak effect against Caco-2 cell line. In comparison to the ethyl acetate derivative **8**, the amide analog **16** showed 50% reduction in the activity against MCF7, while it was inactive against both Caco-2 and A549. Moreover, the hydroxamic acid derivative **17** exhibited nearly half the activity of the analog **8** against A549, whereas it was less potent against Caco-2. However, the acid hydrazide analog **18** and its corresponding Schiff bases **19a-c** were completely devoid of any cytotoxic potential, when compared with their precursor **8**.

#### Cell cycle analysis using flow cytometry

The cell cycle is a tightly regulated, multistep process that governs cellular proliferation through sequential phases, including G1 (cell growth), S (DNA synthesis), G2 (DNA repair), and M (mitosis), with quiescent cells residing in the G0 phase. Progression through these phases is controlled by checkpoint mechanisms to ensure accurate DNA replication and division. It is well established that many anticancer agents exert their cytotoxic effects by disrupting this progression, leading to arrest at specific phases and ultimately inhibiting cell growth. In the present study, the effect of the most potent cytotoxic compound (analog **11**) on cell cycle progression in A549 lung cancer cells was evaluated using propidium iodide staining and flow cytometry, allowing precise quantification of DNA content and distribution across different phases.

**Fig. 2:**
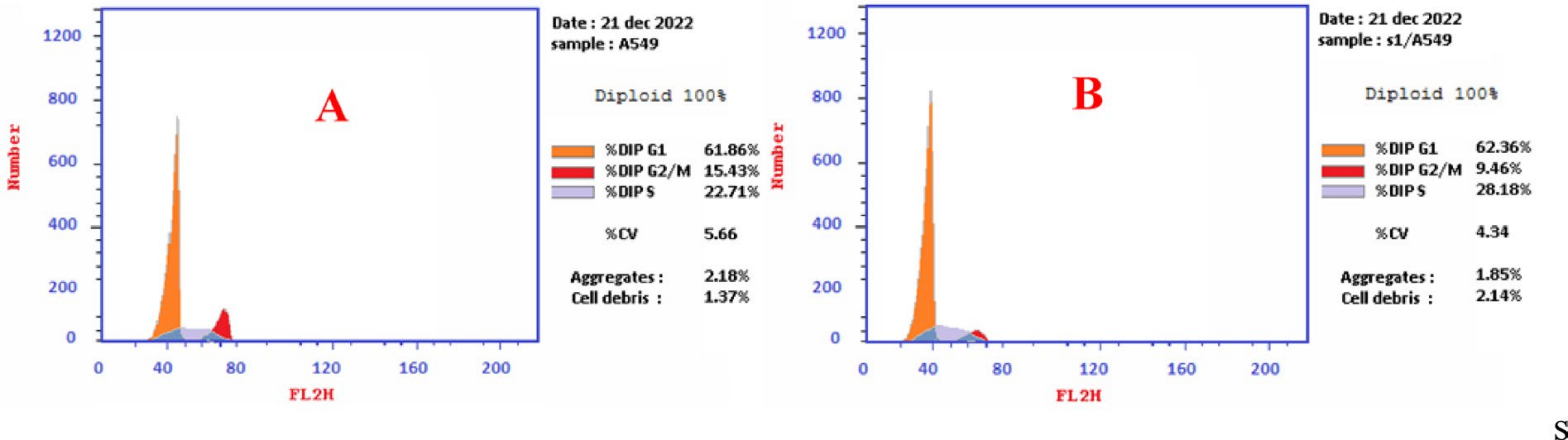
Compound 11 treatment induces S-phase arrest in A549 lung cancer cells. Representative flow cytometric histograms detailing the cell cycle distribution of A549 lung cancer cells under control conditions (Panel A, untreated) and following a 72-h treatment with the IC_50_ concentration (45.12 µM) of Compound 11 (Panel B). Quantitative analysis shows that treatment with Compound 11 leads to a significant quantitative increase in the S-phase cell population to 28.18% (from 22.71% in the untreated control) and a corresponding reduction in the G2/M population to 9.46% (from 15.43% in control), while maintaining a nearly unchanged G0/G1 population. Histograms represent DNA content (PI fluorescence) and the corresponding distribution of cells in G0/G1, S, and G2/M phases, supporting the induction of S-phase arrest.

Following treatment with the IC_50_ concentration (45.12 µM) of compound **11** for 72 h, a clear alteration in cell cycle distribution was observed compared to untreated control cells. As illustrated in Fig. 2, compound 11 significantly increased the proportion of cells in the S phase (28.18%) relative to the control (22.71%), indicating an interruption in DNA synthesis or replication progression. Concurrently, a marked reduction in the G2/M population was detected (9.46% vs. 15.43% in control), suggesting that fewer cells were able to complete DNA replication and enter mitosis. In contrast, the G0/G1 phase remained largely unchanged between treated and untreated groups (62.36% vs. 61.86%), indicating that the compound exerts minimal influence on early cell cycle progression. Collectively, these findings demonstrate that analog **11** effectively induces S-phase cell cycle arrest in A549 cells, thereby suppressing cellular proliferation through interference with DNA replication dynamics.

#### Apoptosis detection utilizing the Annexin V-FITC/PI assay

**Fig. 3:**
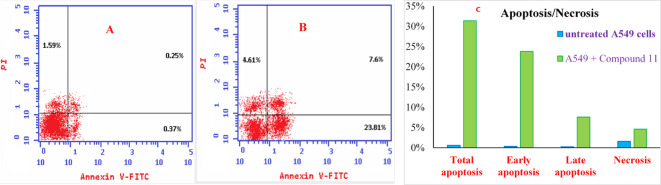
Flow cytometric analysis of apoptosis in A549 cells using Annexin V-FITC/PI dual staining. (**A**) Untreated control cells showing minimal apoptotic and necrotic populations. (**B**) A549 cells treated with compound 11 at its IC_50_ for 72 h, demonstrating a significant increase in early apoptotic (lower right quadrant) and late apoptotic (upper right quadrant) cell populations, with a comparatively minor increase in necrotic cells (upper left quadrant). (**C**) Quantitative representation of total apoptosis, early apoptosis, late apoptosis, and necrosis percentages in untreated and treated cells, confirming apoptosis as the dominant mode of cell death induced by compound 11.

Annexin V-FITC/PI dual staining followed by flow cytometric analysis was employed to elucidate whether the cytotoxic effect of compound **11** on A549 cells is mediated via apoptosis or necrosis. As illustrated in Fig. 3, treatment of A549 cells with compound **11** at its IC_50_ for 72 h resulted in a pronounced shift in cell population toward apoptotic quadrants compared to untreated control cells. Specifically, early apoptotic cells markedly increased to 23.81% versus 0.37% in control cells, while late apoptotic cells rose to 7.6% compared to only 0.25% in untreated cells, corresponding to approximately 64-fold and 30-fold elevations, respectively. In contrast, necrotic cell population exhibited only a modest increase from 1.59% in control cells to 4.61% upon treatment. These findings clearly demonstrate that compound **11** predominantly induces programmed cell death rather than nonspecific necrosis, highlighting apoptosis as the principal mechanism underlying its antiproliferative activity. The preferential activation of both early and late apoptotic pathways aligns with the observed S-phase arrest and supports the mechanistic profile of triazole-based derivatives as effective apoptosis-inducing anticancer agents.

#### Identification of synergism, antagonism, and combination index (CI)

**Fig. 4:**
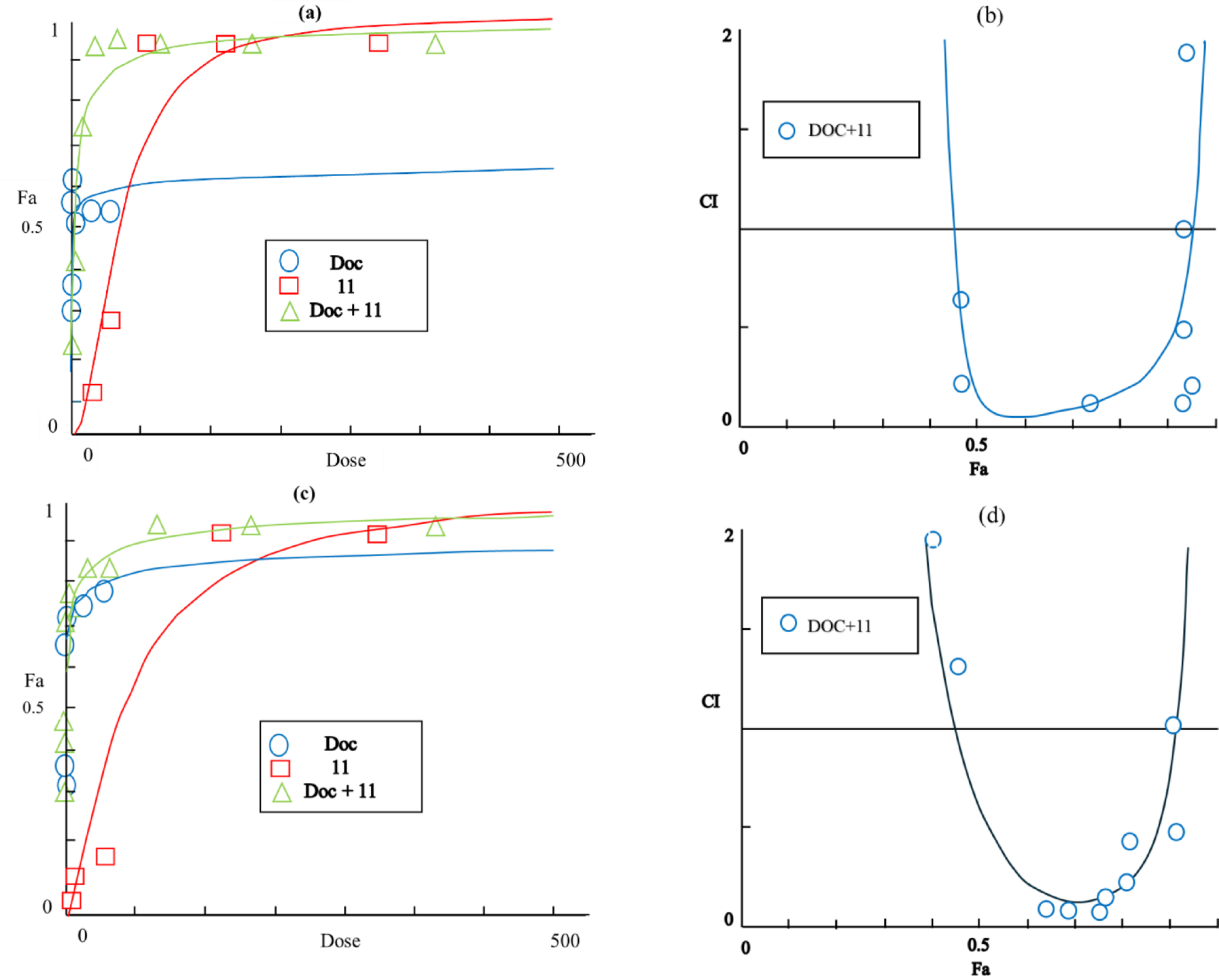
Combination index (CI) analysis of compound **11** with docetaxel in A549 and Caco-2 cancer cells after 72 h treatment. (**a**) Dose–response curves of docetaxel, compound **11**, and their combination in A549 cells showing IC_50_ values. (**b**) CI values as a function of fraction affected (Fa) in A549 cells; CI < 1 indicates synergism. (**c**) Dose–response curves in Caco-2 cells. (**d**) CI vs. Fa plot in Caco-2 cells. Cytotoxicity was assessed by MTT assay, and Fa was calculated as Fa = (% Cell Viability)/100. CI values were derived using CompuSyn software.

The overall purpose of employing the conception of drug combination in chemotherapy is to achieve synergism, improve therapeutic efficacy and response, lower dosage and hence toxicity, and reduce or suppress drug resistance^58,59^. Chou and Talaly developed the term Combination Index (CI) in the 1980s to quantitatively characterize the drug combination impact as synergism (CI < 1), additive effect (CI = 1), or antagonistic effect (CI > 1). For dose–effect assessment, the computer software (CompuSyn) was utilized to estimate the CI and fraction affected-CI (Fa-CI) plot simulation *67*. In this view, the most active broad spectrum cytotoxic member in this study **11** was further investigated as an adjuvant to docetaxel, the standard chemotherapeutic drug. Compound **11** was combined with docetaxel (Doc) at their IC_50_ values to assess the effect of such combination on the inhibition of proliferation of A549 and Caco-2 cells, and the IC_50_ values were estimated for **11** and docetaxel in all combinations. The obtained data presented in Table 5 revealed that the anticancer combination potency of **11** resulted in 87.98% and 43.35% reduction of Doc IC_50_ value against the A549 and Caco-2 cells, respectively. Quantitative analysis of the combination effect using CompuSyn software showed that at the 50% effect level (Fa = 0.5), the CI value was 0.16 for A549 cells and 0.58 for Caco-2 cells. According to established criteria, this corresponds to strong synergism (CI < 0.3) in A549 cells and moderate synergism (0.3 < CI < 0.7) in Caco-2 cells, confirming that the combination of compound **11** and docetaxel produces a synergistic antiproliferative effect, particularly in the A549 cell line. These results were in accordance with previous findings deducing that compounds which halt the cell cycle in the S phase augment the anticancer activity of docetaxel^60,61^. This dual antifungal–anticancer strategy is further supported by recent findings on the immunomodulatory roles of azole derivatives^23^ (Fig. 4).

**Table 5:** IC_50_ (µM) of compound **11** as a single anticancer agent and as an adjuvant to docetaxel (with CI values) against (A) human lung adenocarcinoma A549 (B) Colorectal cancer.

Compound	Doc	11	Doc + 11
A) Adjuvant of Docetaxel (Doc) against A549			
Doc	0.85197	–	–
11	–	49.0421	–
Doc + 11	0.10234	1.94453	0.15978
B) Adjuvant of Docetaxel (Doc) against Caco-2			
Doc	0.06858	–	–
11	–	43.8069	–
Doc + 11	0.03885	0.73821	0.58336

^a)^CI: combination index.

### Molecular docking studies

For further rationalization of the antifungal activity results, a molecular modelling study was conducted to investigate the potential interactions between the promising compound **11** and the active site of lanosterol 14α-demethylase (CYP51). Molecular operating environment software (MOE 2020.09) was utilized on the co-crystal structure of *C. albicans* CYP51 in complex with VT-1161 (PDB code: 5TZ1)^11,62^. The co-crystallized ligand VT-1161 was re-docked to validate the accuracy of our docking protocol (RMSD value = 0.7072).

**Fig. 5:**
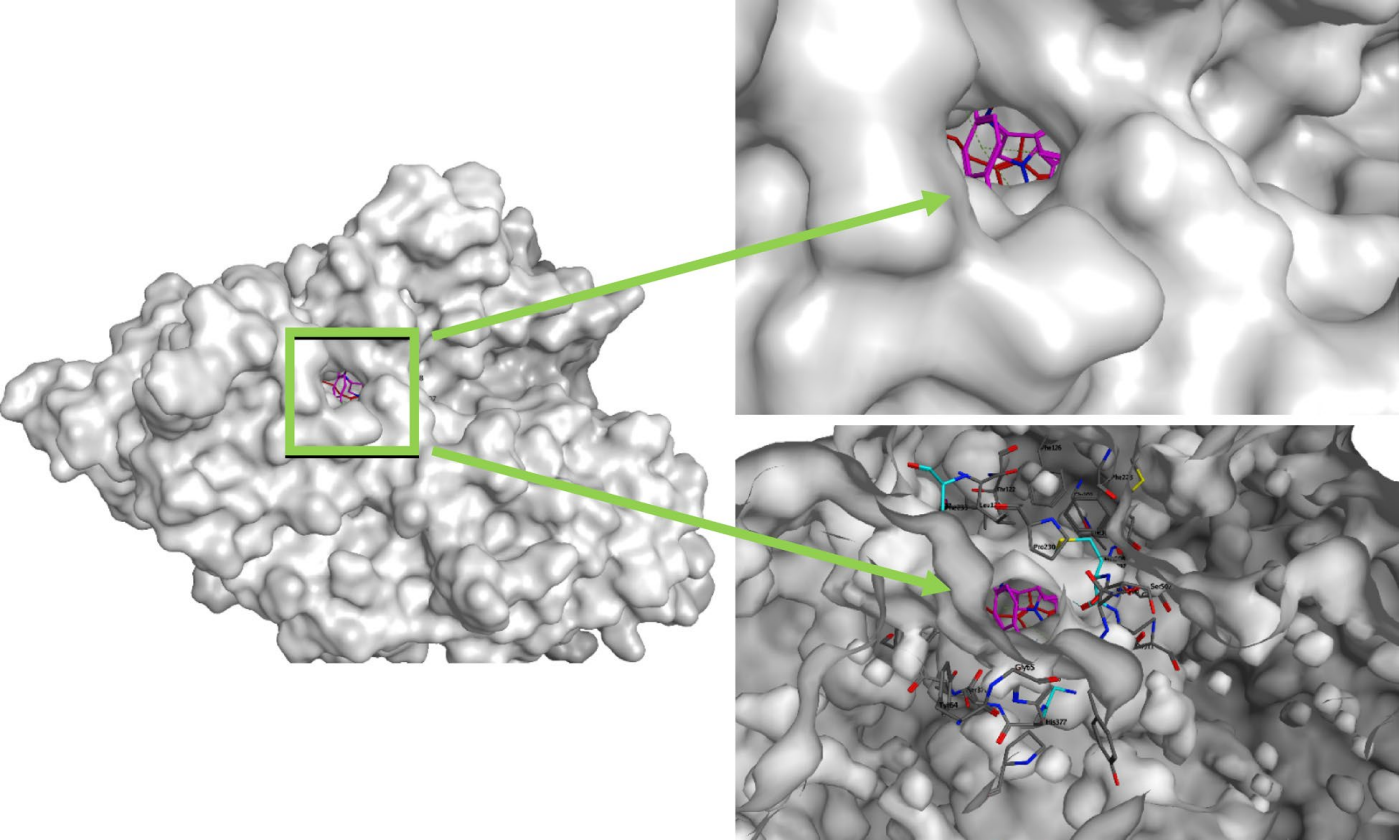
The binding mode of compound **11** in LDM (PDB ID: 5TZ1). (**A**) The cartoon representation of the molecular surface of LDM showing the binding mode of compound 11 in the binding pocket (**B**) Zoomed-in view of LDM active site, (**C**) Transparent mode for the cartoon representation of the enzyme showing the interaction with amino acids involved in the binding pocket.

As shown in Fig. 5, The investigated compound was well penetrated and positioned into the binding site of LDM and was stabilized by a number of polar and hydrophobic molecular interactions, according to molecular modelling analysis. The above-mentioned compound’s binding site residues and overall binding pattern were found to be nearly identical to those observed with the reported ligand (VT-1161)^11^. The docking results demonstrated that compound **11** had moderate binding affinities for LDM (PDB ID: 5TZ1) compared to VT-1161, as shown by the binding free energies and binding interactions of redocked VT-1611 and compounds **11** in Table 6.

**Table 6:** Binding energies and interactions of **VT-1611** and compound **11** with LDM (PDB ID: 5TZ1) active site.

Compound No	Docking scores (Kcal/mol)	Hydrogen bond		Other interactions	
		Residues involved (distance Å)	Functional group	Residues involved (distance Å)	Functional Group
**VT-1161**	− 10.4822	Gly303 (3.42) Leu376 (3.62) Tyr505 (3.41) Met508 (3.50)	2,4-Fphenyl ring **N** of pyridine ring **CH**_**2**_-CF_3_ pyridine ring	Coordination bond with heme iron (2.2)	N^4^ of tetrazole ring
**11**	− 7.8499	Leu376 (3.95) Met508 (3.30)	N^2^ of triazole ring 4-Clphenyl ring	Coordination bond with heme iron (2.41) Arene-Arene heme 5-ring (3.75) H-Arene Tyr118 (3.58) Arene-H Phe233 (4.40) Arene-H Leu376 (4.32)	N^3^ of triazole ring Triazole ring **CH**_**2**_-O-N 4-Clbenzyl ring 4-Clbenzyl ring

**Fig. 6:**
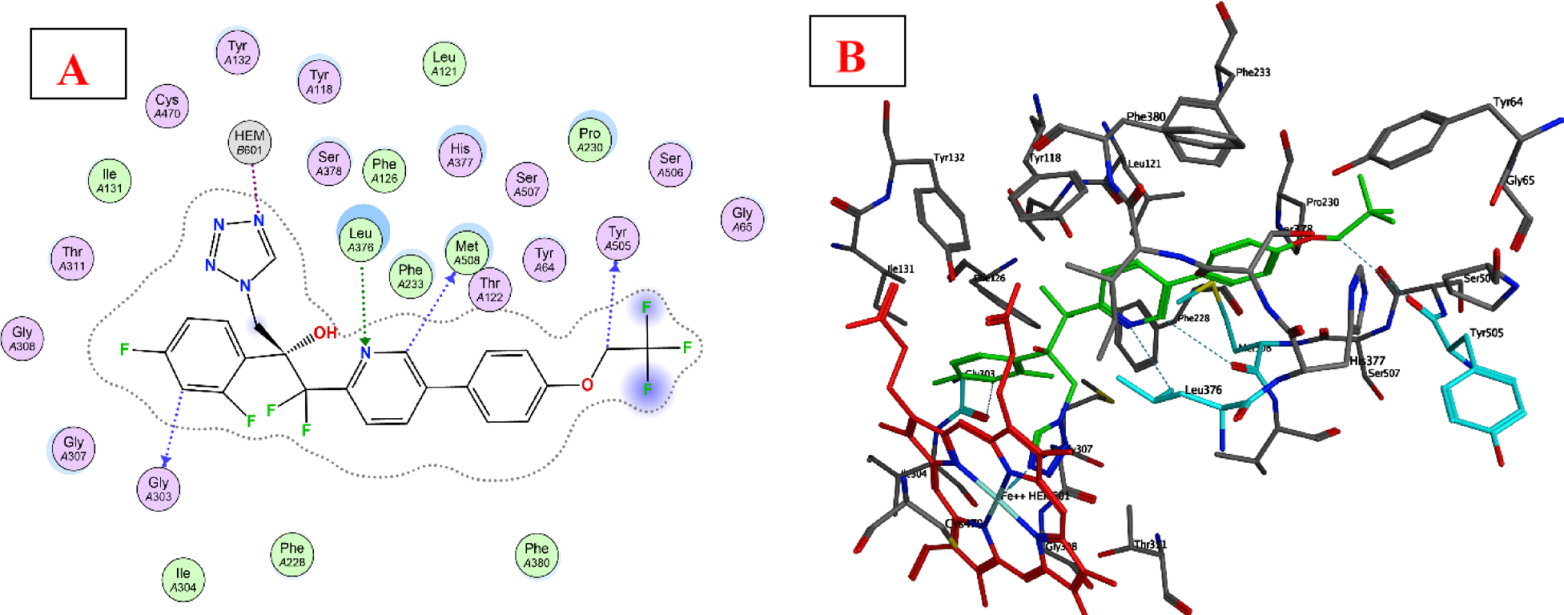
Two-dimensional (**A**) and three-dimensional (**B**) diagrams of co-crystallized ligand **VT-1161** in the active site of CYP51–C. albicans (PDB:5TZ1).

**Fig. 7:**
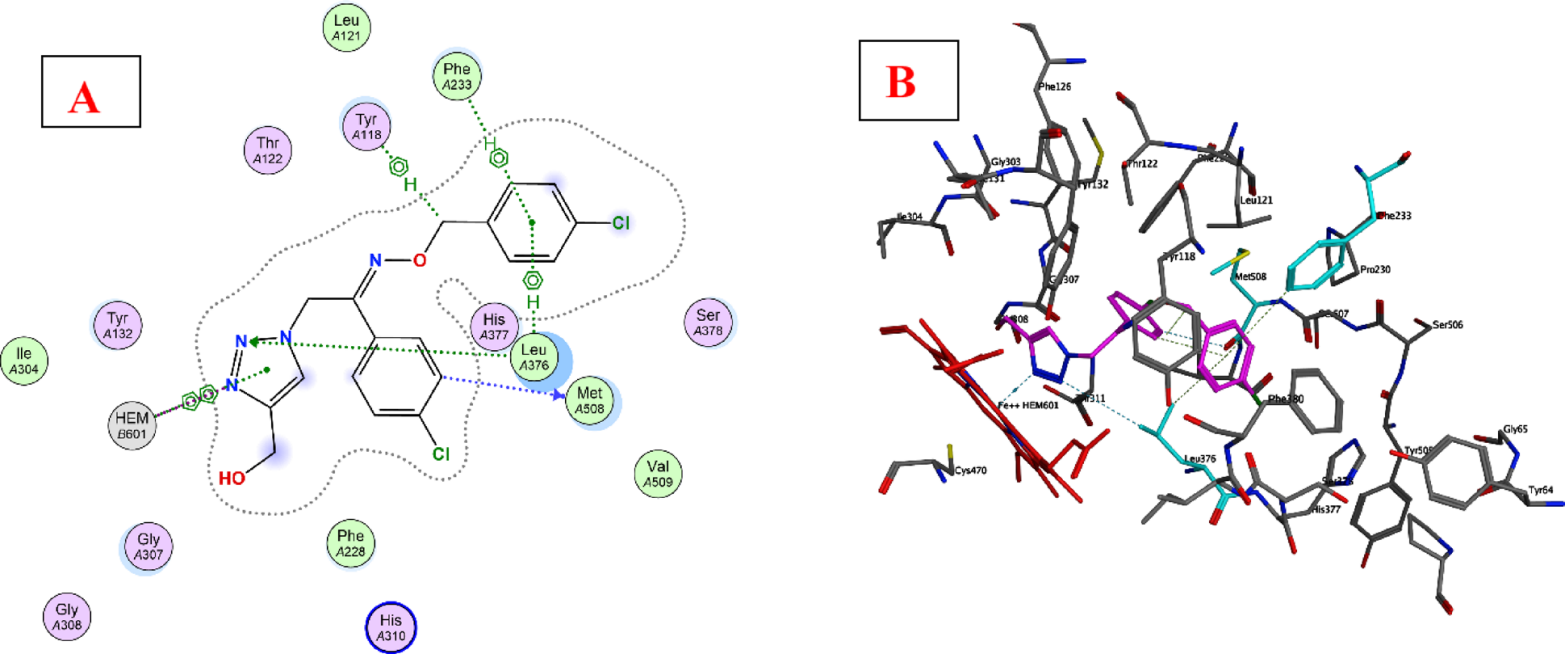
Two-dimensional (**A**) and three-dimensional (**B**) diagrams of compound **11** in the active site of CYP51–C. albicans (PDB:5TZ1).

The triazole ring of compound **11** was linked to the heme group via a coordinate bond between the iron atom and triazole N^3^ atom in addition to pi–pi interaction. Furthermore, the triazole ring had a hydrogen bond as acceptor with Leu 376 via triazole N^2^. The 4-chorobenzyl group occupied the hydrophobic cavity and interacted with the surrounding amino acids Tyr118, Phe233, and Leu376. The other 4-chlorophenyl moiety forms hydrogen bond with Met508, as shown in Fig. 6 and 7. This homology model showed a potential H-Arene interaction with Tyr118, which is a conserved amino acid in *C.albicans-*CYP51, thus supporting the selectivity of the synthesized compound against *C.albicans*^63^*.*

**Fig. 8:**
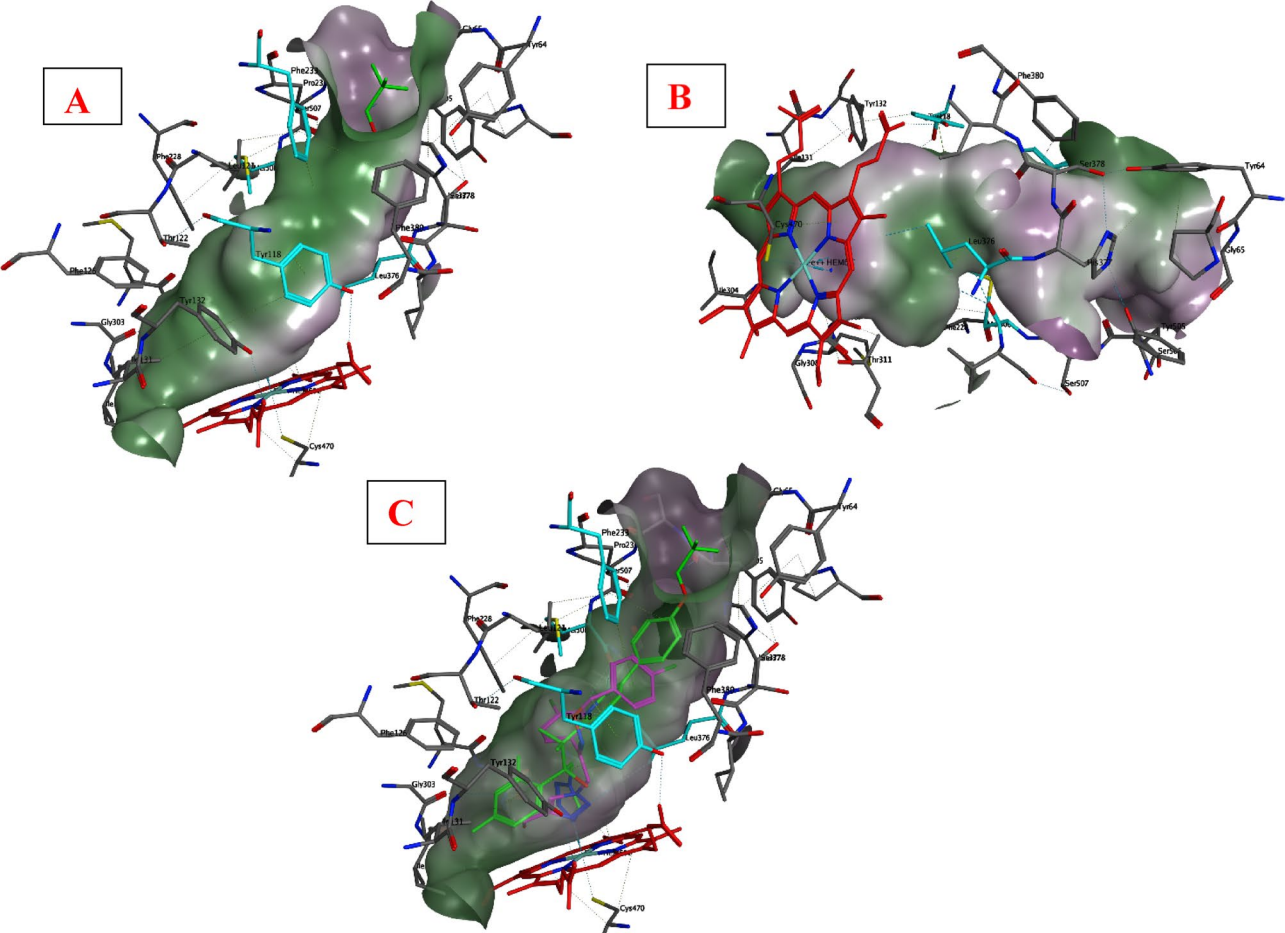
Connolly surface of the active site of LDM bound with compound **11** and **VT-1161**. The binding site surface is colored according to residue hydrophobicity: green-hydrophobic, purple-hydrophilic, and white-neutral. (**A**) Hydrophobic region, (**B**) Hydrophilic region, (**C**) the aromatic rings of both VT-1161 and compound 11 embedded in the hydrophobic region.

**Fig. 9:**
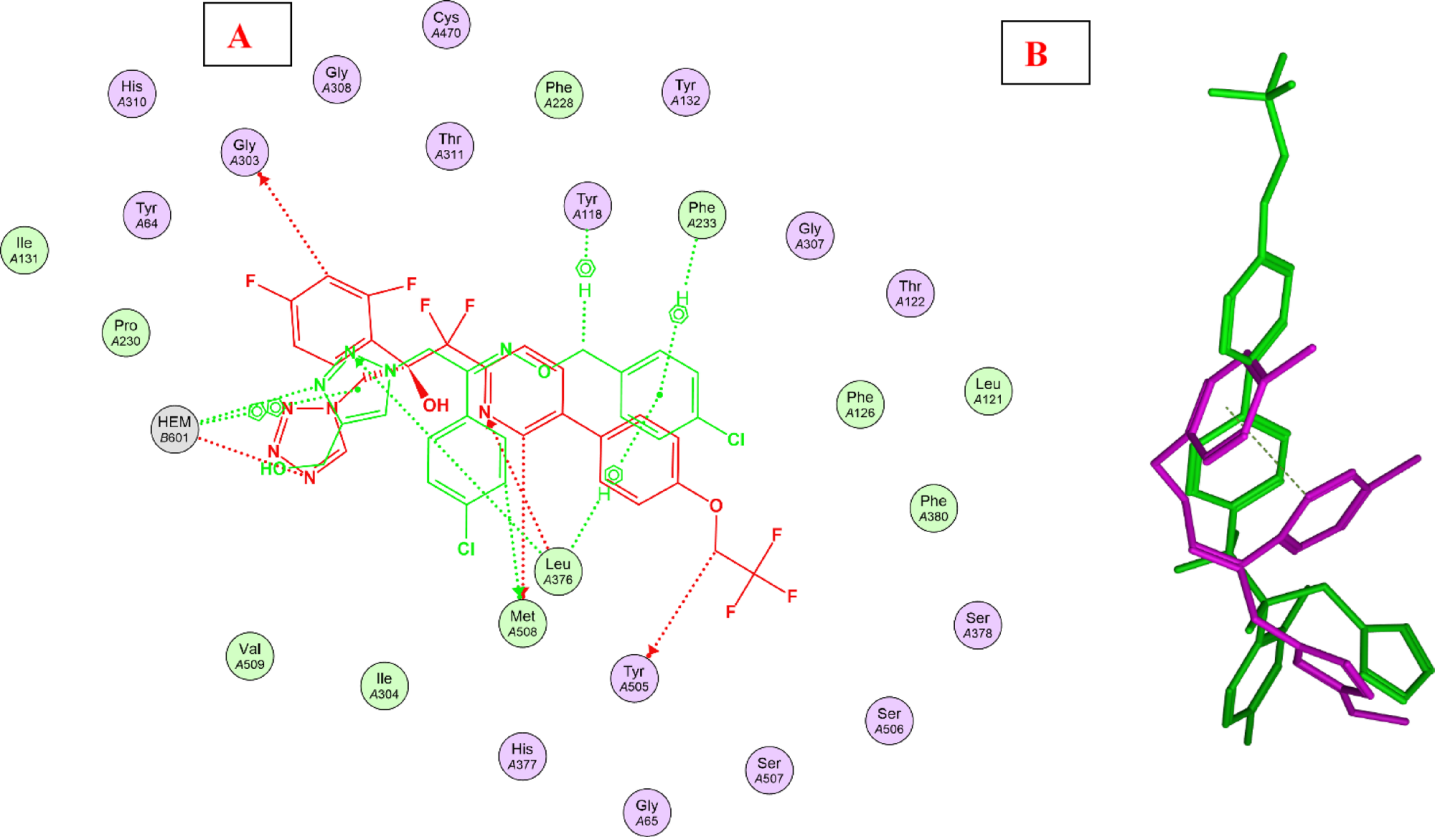
Two-dimensional (**A**) and three-dimensional (**B**) diagrams of superimposition of compound **11** with **VT-1161** in the active site of CYP51–C. albicans (PDB:5TZ1).

The Connolly surface defines the molecular surface, which describes hydrophobic and hydrophilic parts that are responsible for strong pocket tolerance as shown in the binding cavity mapping^64^. The Connolly surface of LDM bound with VT-1161 showed that the 4-chlorophenyl and 4-chlorobenzyl moieties of compound **11** faced the receptor’s hydrophobic area (colored in green), where they underwent hydrophobic interactions with Tyr118, Phe228, Pro230, and Phe233 residues. While the hydrophilic regions of Gly307, Gly308, Leu376, His377, Ser378, Ser506, and Met508 were confronting the hydroxymethyl-1,2,3-triazole moiety and oxyimino functionality. Both aromatic rings were embedded in a hydrophobic pocket at the binding site of the enzyme similar to **VT-1161** as shown in Fig. 8 Moreover, this likeness was supported by the superimposition of compound **11** with **VT-1161** as shown in Fig. 9.

### In-Silico prediction of the physicochemical properties, drug likeness score, pharmacokinetics, and toxicity profile

A key component of the lead optimization phase and the drug development process is the early computation and prediction of the physicochemical properties and the pharmacokinetic profile of new drug candidates, which reduces time, effort, and cost^65,66^. The word "drug-likeness" in this context denotes a balanced combination of a compound’s structure characteristics and variable molecular characteristics to be comparable to previously recognized drugs^67,68^. Several widely used principles as Lipinski’s rule of five (RO5)^67^ and Veber’s criteria^69^ are used to assess the drug-like properties of molecules, such as their hydrophobicity, flexibility (number of rotatable bonds; NROTB), electronic distribution, H-bonding capacity, and molecular size^70,71^. These factors would influence the molecule’s behavior in a biological system, such as bioavailability, membrane transport properties, protein affinity, metabolic stability, reactivity, and toxicity^72^. Therefore, using web-based tools like Molsoft^73^, Molinspiration^74^, Pre-ADMET^75^, and Osiris property explorer^76^, the *In-silico* physicochemical properties, drug likeness score, pharmacokinetic parameters, toxicity profile, and ligand efficiency metrics of the most active compounds **5b**, **8**, **11**, and **19c** were assessed.

The data shown in Table 7 demonstrated that all the examined compounds obeyed Lipinski’s rule of five, with Molecular Weights ranging from 352.78 to 444.85 (≤ 500), HBA numbers between 6 and 9 (≤ 10), and HBD atoms 1–2 (≤ 5) except for compound **5b** which have logP > 5 (5.31). Additionally, all the examined compounds agree with Veber’s rule, exhibiting NROTB values of 6–9 (≤ 10) and topographical polar surface areas (TPSAs) between 72.54 and 114.01 A^2^ (≤ 140 A^2^). These findings point to an acceptable degree of molecular flexibility, which should translate into excellent permeability and oral bioavailability. Additionally, all the analyzed compounds displayed an % ABS range of 69.67–83.79%, which indicates significant oral bioavailability. It should be mentioned that TPSA is inversely proportional with % ABS. For instance, the analogue **11** has the highest absorption (83.97%), while the corresponding TPSA for it is the lowest in the sequence (72.54 2). Their solubility values ranged from 15.25 to 914.57 mg/L, which satisfied the solubility standard (> 0.0001 mg/L), indicating that they would likely have adequate oral absorption, bioavailability, and transport characteristics. In regard to the drug-likeness score values, the results showed that all of the examined compounds produced positive attributes ranging from 0.31 to 0.86.

**Table 7:** In silico prediction of the physicochemical properties, drug likeness, pharmacokinetics, and ligand efficiency metrics of compounds **5b, 8, 11, and 19c.**

		5b	8	11	19c	Oxiconazole
Molinspiration	Physicochemical parameters and drug likeness					
	LogP^a^	5.31	1.84	3.87	2.84	5.65
	M.Wt^b^	376.25	352.78	391.26	444.85	429.13
	HBA^c^	6	8	6	9	4
	HBD^d^	2	1	1	2	0
	Lipinski’s violation^e^	1	0	0	0	1
	NROTB^f^	6	9	7	9	6
	TPSA^g^	75.34	98.85	72.54	114.01	39.42
	%ABS^h^	83.01	74.90	83.97	69.67	95.40
	Volume^i^	308.27	298.04	321.65	367.70	328.06
Molsoft	S^j^	19.23	914.57	15.25	235.77	0.24
	Drug likeness score	0.66	0.31	0.31	0.86	0.44
Pre-ADMET	Pharmacokinetics (ADME)					
	Caco-2^k^	18.01	13.66	21.38	15.23	0.46
	MDCK^l^	0.40	1.69	0.05	0.33	0.04
	HIA^m^	95.30	95.91	96.52	95.49	98.56
	BBB^n^	0.62	0.12	0.13	0.01	0.46
	PPB^o^	97.98	77.24	91.75	90.54	93.10

^a^ *n*-Octanol and water partition coefficient.

^b^ Molecular weight.

^c^ Number of H-bond acceptors.

^d^ Number of H-bond donors.

^e^ Lipinski’s rule of 5 violations (log P ≤ 5, M.Wt. ≤ 500, HBA ≤ 10 and HBD ≤ 5).

^f^ Number of rotatable bonds (Veber’s criteria ≤ 10).

^g^ Topological polar surface area (Veber’s criteria ≤ 140 Å^2^).

^h^ Percentage of absorption = 109 – (0.345*TPSA).

^I^ Molecular volume.

^j^ Solubility in mg/L.

^k^ Permeability through human colon adenocarcinoma cells.

^l^ Permeability through Madin-Darby canine kidney cells.

^m^ Human intestinal absorption percentage.

^n^ Blood brain barrier penetration coefficient.

^o^ Plasma protein binding.

ADME properties were also computed, and the values of the following parameters were determined and compared to their optimal values: Caco-2 cell permeability coefficient, Madin-Darby canine kidney (MDCK) cell permeability coefficient, human intestinal absorption (HIA), blood brain barrier (BBB) coefficient, and plasma protein binding (PPB). The results, which are presented in Table 7, revealed that all of the compounds under investigation had moderate cell permeability in the Caco-2 cell model (13.66–21.38 nm/s) compared to the acceptable range (4–70 nm/s). However, in comparison to the suggested range (25–500 nm/s), all of them showed poor permeability in the MDCK cell model (0.05–1.69 nm/s). In terms of the HIA, the examined compounds had high HIA values (94.56%–96.52%) and were efficiently absorbed. Compounds **8**, **11**, and **19c** exhibited very low BBB permeability coefficients (reported 0.01–0.13), whereas the analogue **5b** displayed moderate permeability (0.62). Except for compound **8**, which exhibited moderate binding to plasma proteins (77.24%), all of the tested compounds were found to be strongly bound to plasma proteins (> 90%), ranging between 90.56% and 97.98%.

Additionally, using Osiris property explorer, the toxicity prediction of the most promising compounds was assessed in terms of mutagenicity, tumorigenicity, irritation, and risk of reproductive effect. All tested compounds lacked any predictable toxicity as mutagenic, tumorigenic, reproductive (except for compound **5b** showed moderate reproductive toxicity), or irritant effects, as shown in Table 8.

**Table 8:** *Toxicity prediction* of compounds **5b, 8, 11, and 19c.**

Osiris property explorer	Toxicity			
Compound No	Mutagenic	Tumorigenic	Reproductive effect	Irritant effect
5b	None	None	moderate	None
8	None	None	None	None
11	None	None	None	None
19c	None	None	None	None

## Conclusion

This work is intended to investigate 1,2,3-triazole compounds inspired by the azole antifungal pharmacophore as possible antifungal agents with additional anticancer effect. Several compounds in the synthesized series showed detectable in vitro antifungal activities against *Candida albicans*, primarily against fluconazole-sensitive strains, demonstrating that azole-related structural characteristics can be incorporated into this structure in order to maintain antifungal activity. Structure–activity patterns showed that the type of substituents linked to the keto and/or oxime moieties had a significant impact on antifungal potency.

Analog **11**, which is structurally similar to oxiconazole, was shown to be the most persistently effective antifungal candidate among the compounds tested. In addition to docking data that indicate favorable positioning within the CYP51 active site, its capacity to suppress ergosterol manufacture in both fluconazole-sensitive and -resistant C. *albicans* strains provides credibility to lanosterol 14α-demethylase inhibition as a likely primary mechanism of action. Although fluconazole combination tests only showed additive or indifferent interactions rather than synergy, compound **11’s** observed additive effect against a resistant strain suggests that it may still be helpful in situations involving resistance even if it does not directly reverse azole resistance.

Simultaneously, some compounds showed moderate cytotoxic action against human cancer cell lines in vitro. Significantly, this activity varied throughout the series, suggesting that antifungal potency does not always correspond to anticancer effectiveness. Also noteworthy was compound **11**, which enhanced the antiproliferative impact of docetaxel in vitro by causing S-phase cell cycle arrest and death in A549 cells. Combination index analysis revealed that this adjuvant effect was synergistic, with strong synergism (CI = 0.16) in A549 cells and moderate synergism (CI = 0.58) in Caco-2 cells at the 50% effect level. These results support a detectable antiproliferative effect, although they do not yet identify a specific molecular target in cancer cells or tumor selectivity.

Taken together, the findings suggest that compound **11** serves as a confirmed in vitro antifungal lead with enhanced antiproliferative activity, suggesting a dual-acting treatment. The current findings support future optimization while also highlighting major shortcomings that must be addressed. Future research should concentrate on selectivity profiling against normal mammalian cells, in vivo efficacy and toxicity assessment, and pharmacokinetic characterization to determine translational potential. In the antifungal setting, resistance evolution studies, and expanded testing against non-*albicans* *Candida* and other pathogenic fungi will be required to characterize the extent and duration of efficacy. Together, these next steps will determine whether oxiconazole-inspired 1,2,3-triazoles can progress from promising in vitro scaffolds to clinically meaningful antifungal or anticancer leads.

## Experimental

### Chemistry

The starting materials and reagents were purchased from Sigma-Aldrich or Merck while.

solvents were purchased from El-Gomhoria Company. Melting points were determined in open-glass capillaries using SMP10—Barloworld scientific melting point apparatus and are not corrected. Follow up of the reactions rates were performed by thin-layer chromatography (TLC) on silica gel (60 GF254) coated glass plates and the spots were visualized by exposure to iodine vapors or UV-lamp at λ 254 nm for few seconds. Infrared spectra (IR) were recorded using KBr discs, frequency ν in (cm^-1^), on PerkinElmer 1430 infrared spectrophotometer, Central Laboratory, Faculty of Pharmacy, Alexandria University, Alexandria, Egypt. Proton nuclear magnetic resonance spectra (^1^H-NMR) and proton decoupled (^13^C-NMR) were scanned on Bruker spectrophotometers (400 MHz) at Microanalytical Unit, Faculty of Pharmacy, Mansoura University, Mansoura, Egypt, and JEOL-ECA II spectrophotometers (500 MHz) at Nuclear Magnetic Resonance unit, Faculty of Science, Mansoura University. Chemical shifts are expressed as δ values (ppm) using tetramethyl silane (TMS) as internal reference. Microanalyses were performed on Vario El Fab-Nr elemental analyzer at Faculty of Pharmacy, Al-Azhar University, Cairo, Egypt.

#### 2-azido-1-(4-chlorophenyl) ethan-1-one (*3*)

To a solution of 4-chlorophenacyl bromide (2.335 g, 10 mmol) dissolved in acetone (20 ml), sodium azide (0.78 g,12 mmol) dissolved in water (20 ml) was added. The solution was stirred for 3 h at room temperature, after which it was poured into ice-water. A yellow ppt was obtained after filtration. Yield: 89%, m.p.: 73–75 °C (reported m.p.: 70–72 °C^77^).

#### 1-(4-chlorophenyl)-2-(4-(hydroxymethyl)-1H-1,2,3-triazol-1-yl) ethan-1-one (*4*)

To a solution of **3** (0.98 gm, 5 mmol) and propargyl alcohol (0.336 g, 6 mmol) in THF (16 ml), CuSO_4_.5H_2_O (250 g, 1 mmol) and sodium ascorbate (0.5 g, 2.5mmol) dissolved in 16 ml water were added. The mixture was stirred for 1 h at room temperature and poured into ice-water. The obtained solid was filtered, washed with water, dried, and crystallized from ethanol: water (1:1).

White crystals, yield: 92%, m.p.: 161–163 °C (reported m.p.: 152 °C^78^), IR (KBr, ν-cm^-1^): 3371 (OH), 3129, 3091, 2985 (C-H), 1691 (C=O), 1590 (C=C), 1573 (N = N), 821 (C=C–Cl). ^1^H NMR (400 MHz, DMSO-*d*_6_): δ 8.10 (d, *J* = 8.2 Hz, 2H, chlorophenyl-C_2,6_-H), 7.95 (s, 1H, triazole-CH), 7.70 (d, *J* = 8.2 Hz, 2H, chlorophenyl-C_3,5_-H), 6.18 (s, 2H, CH_2_-C=O), 5.28 (t, *J* = 5.7 Hz, 1H, OH, D_2_O exchangeable), 4.59 (d, *J* = 5.7 Hz, 2H, CH_2_-OH). ^13^C NMR (100 MHz, DMSO*-d*_6_): 191.92 (C=O), 148.47 (triazole-C_4_), 139.58 (chlorophenyl-C_4_), 133.35 (chlorophenyl-C_1_), 130.57 (chlorophenyl-C_2,6_), 129.59 (chlorophenyl-C_3,5_), 124.88 (triazole-C_5_), 56.21 and 55.55 (CH_2_-OH, CH_2_-C=O). Microanalysis %: Calcd for C_11_H_10_ClN_3_O_2_ (251.67): C, 52.50; H, 4.01; Cl, 14.09; N, 16.70; O, 12.71. Found: C, 52.68%; H, 4.27%; N, 16.93%

#### 1-(2-(4-chlorophenyl)-2-(2-(4-subsituted phenylhydrazineylidene) ethyl)-1H-1,2,3-triazol-4-yl) methanol (5a-c)

To a solution of **4** (0.25 g, 1 mmol) and the appropriate phenylhydrazine hydrochloride (1.1 mmol) in ethanol (5 ml), piperidine (10 drops) was added. The solution was heated under reflux for 9 h, filtered while hot, then poured into ice-water and acidified with dil. HCl. The brown precipitate obtained was filtered, washed with water, and crystallized from ethanol: water (1:2).

##### 1-(2-(4-chlorophenyl)-2-(2-phenylhydrazineylidenethyl)-1H-1,2,3-triazol-4-yl) methanol (5a)

Brown solid, yield: 50%, m.p.: 135–137 °C. IR (KBr, ν-cm^-1^): 3418 (OH), 3145, 3094, 2947 (C-H), 1698 (C=N), 1592 (C=C), 1575 (N = N), 833 (C=C–Cl). ^1^H NMR (500 MHz, DMSO-*d*_6_): δ 13.14 (s, 1H, NH), 8.56 (s, 1H, Triazole-CH), 8.04 & 7.90 (d, *J* = 8.4 , 8.2 Hz, 2H, chlorophenyl-C_2,6_-H, E & Z isomers), 7.65 & 7.53 (d, *J* = 8.4, 8.3 Hz, 2H, chlorophenyl-C_3,5_-H, E & Z isomers), 7.47–7.04 (m, 5H, phenyl-C_2’,3’_,_4’,5’,6’_-H), 6.12 (s, 1H, OH, D_2_O exchangeable), 4.52 (d, *J* = 4.3, 2H, CH_2_-OH), 3.52 (s, 2H, CH_2_-C=N). ^13^C NMR (125 MHz, DMSO-*d*_6_): δ 167.01 (C=N), 148.51 (triazole-C_4_), 139.62 (chlorophenyl-C_4_), 138.34 (phenyl-C_1’_), 133.41 (chlorophenyl-C_1_), 131.70 (chlorophenyl-C_3,5_), 130.64, 130.16 (chlorophenyl-C_3,5_), 129.65 (phenyl-C_4’_), 129.30 (phenyl-C_3’,5’_), 124.93 (triazole-C_5_), 113.68 (phenyl-C_2’,6’_), 55.59 (CH_2_-OH), 44.13 (CH_2_-C=N).Microanalysis %: Calcd C_17_H_16_ClN_5_O (341.80): C, 59.74; H, 4.72; Cl, 10.37; N, 20.49. Found: C, 59.86%; H, 4.97%; N, 20.71%.

##### 1-(2-(4-chlorophenyl)-2-(2-(4-chlorophenyl)hydrazineylidene)ethyl)-1H-1,2,3-triazol-4-yl)methanol (5b)

Brown solid, yield: 60%, m.p.: 148–150 °C. IR (KBr, ν-cm^-1^): 3417 (OH), 3150, 3095, 2933 (C-H), 1699 (C=N), 1591 (C=C), 1574 (N = N), 821 (C=C–Cl). ^1^H NMR (400 MHz, DMSO-*d*_6_): δ 10.51 (s, 1H, NH), 8.04–7.86 (m, 3H, triazole-CH, chlorophenyl-C_2,6_-H), 7.56 (d, *J* = 8.50, Hz 2H, chlorophenyl-C_3,5_-H), 7.47–7.15 (m, 4H, chlorophenyl-C_2’,3’,5’,6’_-H), 5.82 (s, 2H, CH_2_-C=N), 5.16 (s, 1H, OH, D_2_O exchangeable ), 4.48 (s, 2H, CH_2_-OH). ^13^C NMR (125 MHz, DMSO-*d*_6_): δ 144.52 (C=N), 140.65 (triazole-C_4_), 139.63 (chlorophenyl-C_1’_), 135.39 (chlorophenyl-C_4_), 133.06 (chlorophenyl-C_1_), 130.63,129.65 (chlorophenyl-C_2,3,5,6_), 129.00 (chlorophenyl-C_3’,5’_), 127.51 (chlorophenyl-C_4’_), 123.98 (triazole-C_5_), 117.45, 115.15 (chlorophenyl-C_2’,6’_), 55.45 (CH_2_-OH), 43.19 (CH_2_-C=N). Microanalysis %: Calcd C_17_H_15_Cl_2_N_5_O (376.24): C, 54.27; H, 4.02; Cl, 18.84; N, 18.61. Found: C, 54.61%; H, 4.25%; N, 18.75%.

##### 1-(2-(4-chlorophenyl)-2-(2-(4-fluorophenyl)hydrazineylidene)ethyl)-1H-1,2,3-triazol-4-yl) methanol (5c)

Brown solid, yield: 60%, m.p.: 145–147 °C. IR (KBr, ν-cm^-1^): 3408 (OH), 3266 (NH), 3124, 3078, 2856 (C-H), 1690 (C=N), 1596 (C=C), 1581 (N = N), 1210 (C-F), 826 (C=C–Cl). ^1^H NMR (400 MHz, DMSO-*d*_*6*_): δ 10.34 (s, 1H, NH, D_2_O exchangeable), 7.93 (s, 1H, triazole-CH), 7.81 (d, *J* = 8.5 Hz, 2H, chlorophenyl-C_2,6_-H), 7.42 (d, *J* = 8.6 Hz, 2H, chlorophenyl-C_3,5_-H), 7.32–7.24 (m, 2H, fluorophenyl-C_2’,6’_-H), 7.14 (t, *J*_*H,F*_ = 8.8 Hz, *J* = 8.9 Hz, 2H, fluorophenyl-C_3’,5’_-H), 5.79 (s, 2H, CH_2_-C=N), 5.15 (t, J = 5.7 Hz, 1H, OH), 4.47 (d, J = 5.7 Hz, 2H, CH_2_-OH). ^13^C NMR (125 MHz, DMSO-*d*_6_): δ 158.10, 156.23 (J = 233 Hz, fluorophenyl-C_4’_), 148.75 (triazole-C_4_), 142.17 (C=N), 136.28 (chlorophenyl-C_4_), 134.53 (fluorophenyl-C_1’_), 132.84 (chlorophenyl-C_1_), 130.64, 129.71, 128.98, 127.39 (chlorophenyl-C_2,3,5,6_), 123.45 (triazole-C_5_), 116.34 & 116.16 (J = 22.5 Hz, fluorophenyl-C_3’,5’_), 114.79 & 114.73 (J = 7.5 Hz, fluorophenyl-C_2’,6’_), 55.44 (CH_2_-OH), 43.13 (CH_2_-C=N). Microanalysis %: Calcd C_17_H_15_ClFN_5_O (359.79): C, 56.75; H, 4.20; Cl, 9.85; F, 5.28; N, 19.47; O, 4.45. Found: C, 57.02%; H, 4.31%; N, 19.70%.

#### 1-(4-chlorophenyl)-2-(4-(hydroxymethyl)-1H-1,2,3-triazol-1-yl) ethan-1-one oxime (*6*)

To a mixture of** 4** (0.25 g, 1 mmol) and hydroxylamine HCl (0.08 g, 1.2 mmol) in ethanol, (5 ml) sodium acetate (0.1 g, 1.2 mmol) was added. The solution was heated under reflux for 3 h, then filtered while hot and the filtrate was concentrated. The formed crystals were filtered, washed with cold ethanol, dried, and recrystallized from ethanol.

White crystals, yield: 75%, m.p.: 156–158 °C. IR (KBr, ν-cm^-1^): 3236 (OH), 3146, 3045, 2879 (C-H), 1620 (C=N), 1595 (C=C), 1567 (N = N), 837 (C=C–Cl). ^1^H NMR (400 MHz, DMSO-*d*_6_): δ 12.24 (s, 1H, C=NOH, D_2_O exchangeable), 7.89 (s, 1H, triazole-CH), 7.75 (d, *J* = 8.5 Hz, 2H, chlorophenyl-C_2,6_-H), 7.48 (d, *J* = 8.4 Hz, 2H, chlorophenyl-C_3,5_-H), 5.69 (s, 2H, , CH_2_-C=N), 5.18 (t, *J* = 5.7 Hz, 1H, OH, D_2_O exchangeable), 4.47 (d, *J* = 5.7 Hz, 2H, CH_2_-OH). ^13^C NMR (125 MHz, DMSO-*d*_6_): δ 150.43 (C=N), 148.56 (triazole-C_4_), 134.47 (chlorophenyl-C_4_), 133.52 (chlorophenyl-C_1_), 130.92 (chlorophenyl-C_2,6_), 129.13,128.40 (chlorophenyl-C_3,5_), 123.94 (triazole-C_5_), 55.44 (CH_2_-OH), 42.95 (CH_2_-C=N).Microanalysis %: Calcd for C_11_H_11_ClN_4_O_2_ (266.68): C, 49.54; H, 4.16; Cl, 13.29; N, 21.01. Found: C, 49.80%; H, 4.29%; N, 21.28%.

#### 2-(1-(4-chlorophenyl)-2-(4-(hydroxymethyl)-1H-1,2,3-triazol-1-yl) ethylidene)hydrazine-1-carbothioamide (*7*)

To a solution of **4** (0.25 g, 1 mmol) and thiosemicarbazide (0.1g, 1.1 mmol) in ethanol (5 ml), piperidine (10 drops) was added. The solution was heated under reflux for 7 h, filtered while hot, then poured into ice-water and acidified using dil. HCl. The obtained solid was filtered, washed with water, dried, and crystallized from ethanol: water (1:2).

Yellow solid, yield: 35%, m.p.: 173–175 °C. IR (KBr, ν-cm^-1^): 3371 (broad band OH, NH_2_), 3309 (NH), 3175, 2973, 2924 (C-H), 1633 (C=N), 1587 (C=C), 1568 (N = N), 1276 (C=S), 837 (C=C–Cl). ^1^H NMR (500 MHz, DMSO-*d*_6_): δ 13.26 (s, 1H, C=N–NH), 7.93–7.86 (m, 3H, chlorophenyl-C_2,6_-H, triazole-CH), 7.86 and 7.61 (2d, *J* = 8.5, 8.5 Hz, 1H each, S = C-NH_2_), 7.53 (d, *J* = 8.7 Hz, 2H, chlorophenyl-C_3,5_-H), 4.13 (t, *J* = 5.5 Hz, 1H, OH, D_2_O exchangeable), 3.47 (d, *J* = 5.3 Hz, 2H, CH_2_-OH), 3.37 ( s, 2H, CH_2_-C=N) in the range of H_2_O of ^1^H-NMR but appeared in the D_2_O exchange chart. ^13^C NMR (125 MHz, DMSO-*d*_6_): δ 186.45 (C=S), 167.02 (C=N–NH-C=S), 139.92 (triazole-C_4_), 138.34 (chlorophenyl-C_4_), 131.68 (chlorophenyl-C_1_), 130.12 (chlorophenyl-C_3,5_), 129.88 (triazole-C_5_), 129.28 (chlorophenyl-C_2,6_), 53.16 (CH_2_-OH), 48.10 (CH_2_-C=N). Microanalysis %: Calcd for C_12_H_13_ClN_6_OS (324.79): C, 44.38; H, 4.03; Cl, 10.91; N, 25.88. Found: C, 44.60%; H, 4.20%; N, 25.72%.

#### Ethyl 2-(((1-(4-chlorophenyl)-2-(4-(hydroxymethyl)-1H-1,2,3-triazol-1-yl)ethylidene)-amino)oxy)acetate (*8*)

To a mixture of **6** (0.27 g, 1 mmol) and anhydrous potassium carbonate (0.21 g, 1.5 mmol) in acetonitrile (5ml), ethyl bromoacetate (0.18 g, 1.1 mmol) was added dropwise. The mixture was stirred at room temperature for 24 h, filtered, and the filtrate was left to evaporate. The obtained solid was triturated with methylene chloride, filtered and the filtrate was evaporated under reduced pressure leaving a white solid that was crystallized from ethanol: water (1:2).

White crystals, yield: 90%, m.p.: 110–112 °C. IR (KBr, ν-cm^-1^): 3438 (OH), 3133, 2986, 2935 (C-H), 1741 (C=O), 1741 (C=N), 1592 (C=C), 1560 (N = N),1215 and 1103 (C–O–C), 847 (C=C–Cl). ^1^H NMR (400 MHz, DMSO-*d*_6_): δ 8.00 (s, 1H, triazole-CH), 7.71–7.69 (d, 2H, *J* = 8.7 Hz, chlorophenyl-C_2,6_-H), 7.50–7.48 (d, 2H, *J* = 8.6 Hz, chlorophenyl-C_3,5_-H), 5.78 (s, 2H, CH_2_-C=N), 5.16 (t, *J* = 5.7 Hz, 1H, OH, D_2_O exchangeable), 4.93 (s, 2H, CH_2_-C=O), 4.47 (d, *J* = 5.6 Hz, 2H, CH_2_-OH), 4.19 (q, *J* = 7.0 Hz, 2H, O-CH_2_-CH_3_), 1.22 (t, *J* = 7.1 Hz, 3H, O-CH_2_-CH_3_). Microanalysis %: Calcd for C_15_H_17_ClN_4_O_4_ (352.78): C, 51.07; H, 4.86; Cl, 10.05; N, 15.88. Found: C, 51.35%; H, 4.98%; N, 16.04%.

#### 1-(4-chlorophenyl)-2-(4-(hydroxymethyl)-1H-1,2,3-triazol-1-yl) ethan-1-one O-ethoxycarbonyl oxime (*9*)

To a mixture of **6** (0.27 g, 1 mmol), spikes of potassium iodide, and anhydrous potassium carbonate (0.21 g, 1.5 mmol) in acetonitrile (5ml), ethyl chloroformate (0.12 g, 0.1 ml, 1.1 mmol) was added dropwise. The mixture was stirred at room temperature for 2 h, filtered, and the filtrate was left to evaporate. The obtained solid was triturated with methylene chloride, filtered and the filtrate was evaporated under reduced pressure leaving a pale-yellow solid that was crystallized from ethanol: water (1:2).

Pale-yellow crystals, yield: 70%, m.p.; 73–75 °C. IR (KBr, ν-cm^-1^): 3382 (OH), 3148, 2983, 2934 (C-H), 1746 (C=O), 1633 (C=N), 1594 (C=C), 1568 (N = N),1261, 1093 (C–O–C), 837 (C=C–Cl). ^1^H NMR (500 MHz, DMSO-*d*_*6*_): δ 8.09 (s, 1H, triazole-CH), 7.68 (d, *J* = 8.4 Hz, 2H, chlorophenyl-C_2,6_-H), 7.41 (d, *J* = 8.2 Hz, 2H, chlorophenyl-C_3,5_-H), 5.65 (s, 2H, CH_2_-C=N), 5.16 (s, 1H, OH**,** D_2_O exchangeable), 5.07 (d, *J* = 7.3 Hz, 2H, CH_2_-OH), 4.06 (q, *J* = 7.2 Hz, 2H, O-CH_2_-CH_3_), 1.14 (t, *J* = 7.2 Hz, 3H, O-CH_2_-CH_3_). ^13^C NMR (125 MHz, DMSO-*d*_6_): δ 154.75 (C=O), 150.31 (C=N), 141.84 (triazole-C_4_), 134.67 (chlorophenyl-C_4_), 133.42 (chlorophenyl-C_1_), 129.11 (chlorophenyl-C_3,5_), 128.42 (chlorophenyl-C_2,6_), 126.42 (triazole-C_5_), 64.32 (O-CH_2_-CH_3_), 60.59 (CH_2_-OH), 43.23 (CH_2_-C=N), 14.57 (O-CH_2_-CH_3_). Microanalysis %: Calcd for C_14_H_15_ClN_4_O_4_ (338.75): C, 49.64; H, 4.46; Cl, 10.47; N, 16.54. Found: C, 49.85%; H, 4.70%; N, 16.73%.

#### 1-(4-chlorophenyl)-2-(4-(hydroxymethyl)-1H-1,2,3-triazol-1-yl) ethan-1-one O-benzyl oxime (*10*)

To a mixture of **6** (0.27 g, 1 mmol) and anhydrous potassium carbonate (0.21 g, 1.5 mmol) dissolved in acetonitrile (5 ml), benzyl bromide (0.17 g, 1 mmol) was added. The mixture was stirred at room temperature for 24 h, filtered, and the filtrate was left to evaporate. The obtained solid was triturated with methylene chloride, filtered and the filtrate was evaporated under reduced pressure leaving a yellow solid that was crystallized from ethanol: water (1:2).

Pale-yellow crystals, yield: 60% m.p.: 70–71 °C. IR (KBr, ν-cm^-1^): 3377 (OH), 3169, 3032, 2940 (C–H), 1633 (C=N), 1598 (C=C), 1565 (N = N), 837 (C=C–Cl). ^1^H NMR (400 MHz, DMSO-*d*_*6*_): δ 7.85 (s, 1H, triazole-CH), 7.70 (d, *J* = 8.2 Hz, 2H, chlorophenyl-C_2,6_-H), 7.46 (d, *J* = 8.2 Hz, 2H, chlorophenyl-C_3,5_-H), 7.41 – 7.30 (m, 5H, phenyl-H), 5.71, 5.30 (2s, each 2H, CH_2_-C=N), 5.20 (t, *J* = 5.7 Hz, 1H, OH, D_2_O exchangeable), 4.46 (d, *J* = 5.6 Hz, 2H, CH_2_-OH). ^13^C NMR (125 MHz, DMSO-*d*_6_): δ 151.70 (C=N), 148.59 (triazole-C_4_), 137.75 (phenyl-C_1’_), 135.11 (chlorophenyl-C_4_), 132.48 (chlorophenyl-C_1_), 129.22 (chlorophenyl-C_3,5_), 128.95 (chlorophenyl-C_2,6_), 128.83 (phenyl-C_2’,6’_), 128.62 (phenyl-C_3’,5’_), 128.49 (phenyl-C_4’_), 124.08 (triazole-C_5_), 76.8 (O-CH_2_), 55.44 (CH_2_-OH), 42.95 (CH_2_-C=N).Microanalysis %: Calcd for C_18_H_17_ClN_4_O_2_ (356.356.81): C, 60.59; H, 4.80; Cl, 9.94; N, 15.70. Found: C, 60.42%; H, 4.96%, N, 15.94%.

#### 1-(4-chlorophenyl)-2-(4-(hydroxymethyl)-1H-1,2,3-triazol-1-yl) ethan-1-one O-(4-chlorobenzyl) oxime (*11*)

To a mixture of **6** (0.27 g, 1 mmol), spikes of potassium iodide, and anhydrous potassium carbonate (0.21 g, 1.5 mmol) in acetonitrile (5 ml), 4-chlorobenzyl chloride (0.16 g, 1 mmol) was added. The mixture was stirred at room temperature for 24 h, filtered, and the filtrate was left to evaporate. The obtained solid was triturated with methylene chloride, filtered and the filtrate was evaporated under reduced pressure leaving a white solid that was crystallized from ethanol: H_2_O (1:2).

White crystals, yield: 65%, m.p.: 75–77 °C. IR (KBr, ν-cm^-1^): 3325 (OH), 3167, 3029, 2940 (C-H), 1727 (C=N), 1596 (C=C), 1492 (N = N), 838 (C=C–Cl). ^1^H NMR (400 MHz, DMSO-*d*_*6*_): δ 7.87 (s, 1H, triazole-CH), 7.70 (d, *J* = 8.2 Hz, 2H, chlorophenyl-C_2,6_-H), 7.46 (d, *J* = 8.3 Hz, 2H, chlorophenyl-C_3,5_-H), 7.41–7.31 (m, 4H, chlorophenyl-C_2’,3’,5’,6’_-H), 5.71, 5.28 (2s, each 2H, CH_2_-C=N), 5.21 (t, *J* = 5.8, 1H, OH, D_2_O exchangeable), 4.46 (d, *J* = 5.7 Hz, 2H, CH_2_-OH). ^13^C NMR (125 MHz, DMSO-*d*_6_): δ 152.08 (C=N), 148.51 (triazole-C_4_), 136.86 (chlorophenyl-C_1’_), 135.17 (chlorophenyl-C_4_), 133.10 (chlorophenyl-C_4’_), 132.43 (chlorophenyl-C_1’_), 130.46 (chlorophenyl-C_3,5_), 129.23 (chlorophenyl-C_3’,5’_), 128.9 (chlorophenyl-C_2,6,2’,6’_), 124.15(triazole-C_5_), 75.84 (O-CH_2_), 55.4(CH_2_-OH), 43.76 (CH_2_-C=N). Microanalysis %: Calcd for C_18_H_16_Cl_2_N_4_O_2_ (391.25): C, 55.26; H, 4.12; Cl, 18.12; N, 14.32. Found: C, 55.43%; H, 4.25%; N, 14.59%.

#### 1-(4-chlorophenyl)-2-(4-(hydroxymethyl)-1H-1,2,3-triazol-1-yl) ethan-1-one O-prop-2-yn-1-yl oxime (*12*)

To a mixture of **6** (0.27 g, 1 mmol) and potassium hydroxide (0.08 g, 1.5 mmol) in DMSO (5 ml), propargyl bromide (0.14 g, 1.2 mmol) was added. The mixture was stirred under reflux for 7 h, filtered, and the filtrate was poured into ice-water. The obtained solid was filtered, triturated with methylene chloride, and filtered. The filtrate was evaporated under reduced pressure leaving a solid that was crystallized from ethanol.

Brown crystals, yield: 40%, m.p.: 72–74 °C. IR (KBr, ν-cm^-1^): 3429 (OH), 3307 (C≡C-H) 2947, 2927, 2858 (C-H), 2037 (C≡C), 1727 (C=N), 1595 (C=C), 1490 (N = N), 837 (C=C–Cl). ^1^H NMR (400 MHz, DMSO-*d*_6_): δ 8.04–7.74 (m, 3H, triazole-CH, chlorophenyl-C_2,6_-H), 7.65 (d, *J* = 8.3 Hz, 2H, chlorophenyl-C_3,5_-H), 4.46 (t, *J* = 7.0 Hz, 1H, OH, D_2_O exchangeable), 4.18 (s, 2H, CH_2_-C=N), 3.69–3.47 (m, 4H, CH_2_-OH, CH_2_-C≡CH), 3.00 (s, 1H, C≡C-H). Microanalysis %: Calcd for C_14_H_13_ClN_4_O_2_ (304.73): C, 55.18; H, 4.30; Cl, 11.63; N, 18.39. Found: C, 55.40%; H, 4.41%; N, 18.64%.

#### 1-(4-chlorophenyl)-2-(4-(hydroxymethyl)-1H-1,2,3-triazol-1-yl) ethan-1-one O-(2-chloroacetyl) oxime (*13*)

To a mixture of **6** (0.27 g, 1 mmol), spikes of potassium iodide and anhydrous potassium carbonate (0.21 g, 1.5 mmol) in acetone (5 ml), chloroacetyl chloride (0.17 g, 1.5 mmol) was added dropwise. The mixture was stirred at room temperature for 12 h, filtered, and the filtrate was left to evaporate. The obtained solid was triturated with methylene chloride, filtered and the filtrate was evaporated under reduced pressure leaving a yellow solid that was crystallized from ethanol: H_2_O (1:2).

Pale yellow crystals, yield: 40%, m.p.: 132–134 °C. IR (KBr, ν-cm^-1^): 3413 (OH), 3161, 3120, 2928 (C-H), 1769 (C=O), 1741 (C=N), 1591 (C=C), 1469 (N = N), 849 (C=C–Cl). ^1^H NMR (500 MHz, DMSO-*d*_6_): δ 8.62 (s, 1H, triazole-C_4_-H), 7.63 (d, *J* = 8.2 Hz, chlorophenyl-C_2,6_-H), 7.43 (d, *J* = 8.2 Hz, 2H, chlorophenyl-C_3,5_-H), 5.81, 5.25 (2 s, each 2H, CH_2_-C=N, CH_2_-Cl), 5.01 (s, 2H, CH_2_-OH ) 4.42 (s, 1H, OH, D_2_O exchangeable). ^13^C NMR (125 MHz, DMSO-*d*_6_): δ 170.66 (C=O), 152.37 (C=N), 143.4 (triazole-C_4_),135.72 (chlorophenyl-C_4_), 131.18 (chlorophenyl-C_1_), 129.29 (chlorophenyl C_3,5_), 128.47 (chlorophenyl-C_2,6_), 126.53 (triazole-C_5_), 71.15 (CH_2_-Cl), 58.35 (CH_2_-OH), 43.30 (CH_2_-C=N). Microanalysis %: Calcd for C_13_H_12_Cl_2_N_4_O_3_ (343.16): C, 45.50; H, 3.52; Cl, 20.66; N, 16.33. Found: C, 45.73%; H, 3.68%; N, 16.5%.

#### 1-(4-chlorophenyl)-2-(4-(hydroxymethyl)-1H-1,2,3-triazol-1-yl) ethan-1-one O-(2-bromoacetyl) oxime (*14*)

To a mixture of **6** (0.27 g, 1 mmol) and anhydrous potassium carbonate (0.21 g, 1.5 mmol) in acetone (5 ml), bromoacetyl bromide (0.3 g, 1.5 mmol) was added dropwise. The mixture was stirred at room temperature for 24 h, filtered, and the filtrate was left to evaporate. The obtained solid was triturated with methylene chloride, filtered and the filtrate was evaporated under reduced pressure leaving a yellow solid that was crystallized from ethanol: H_2_O (1:2).

Pale yellow crystals, yield: 40%, m.p.: 163–165 °C. IR (KBr, CM^-1^): 3417 (OH), 3161, 3120, 2988, 2959 (C-H), 1769 (C=O), 1742 (C=N), 1591 (C=C), 1496 (N = N), 849 (C=C–Cl), 516 (CH_2_-Br). ^1^H NMR (500 MHz, CDCl_3_): δ 8.85, 8.06 (s, 1H, triazole-C-H, E & Z isomers), 7.67, 7.60 (2d, *J* = 8.3, 8.7 Hz, 2H, chlorophenyl-C_2,6_-H, E & Z isomers), 7.31 & 7.27 (2d, *J* = 7.9, 8.8 Hz, 2H, chlorophenyl-C_3,5_-H, E & Z isomers), 5.69, 5.66 (2s, each 2H, CH_2_, E & Z isomers), 5.36, 5.30 (2s, 2H, CH_2_, E & Z isomers), 4.86 (d,* J* = 9.4 Hz, 2H, CH_2_-OH), 4.26 (t, *J* = 7.2 Hz, 1H, OH). Microanalysis %: Calcd for C_13_H_12_Br 2ClN_4_O_3_ (387.62): C, 40.28; H, 3.12; Br, 20.61; Cl, 9.15; N, 14.45. Found: C, 40.47%; H, 3.29%; N, 14.67%.

#### 1-(4-chlorophenyl)-2-(4-(hydroxymethyl)-1H-1,2,3-triazol-1-yl) ethan-1-one O-benzoyl oxime (*15*)

To a mixture of **6** (0.27 g, 1 mmol) and spikes of potassium iodide in acetonitrile (5 ml), benzoyl chloride (0.14 g, 1 mmol) was added. The mixture was stirred at room temperature for 6 h, filtered, and the filtrate was left to evaporate. The obtained solid was triturated with methylene chloride and filtered. The filtrate was evaporated under reduced pressure leaving a yellow solid that was crystallized from ethanol: H_2_O (1:2).

Yellow crystals, yield: 80%, m.p.: 188–190 °C. IR (KBr, ν-cm^-1^): 3407 (OH), 3142, 3015, 2859 (C-H), 1717 (C=O), 1654 (C=N), 1599 (C=C), 1497 (N = N), 838 (C=C–Cl). ^1^H NMR (500 MHz, DMSO-*d*_*6*_): δ 8.19 (s, 1H, triazole-C-H), 7.89, 7.84 (2d, *J* = 7.8, 7.0 Hz, 2H, chlorophenyl-C_2,6_-H, E & Z isomers), 7.68, 7.62 (2d, *J* = 8.7, 7.2 Hz, 2H, chlorophenyl-C_3,5_-H, E & Z isomers), 7.51 – 7.24 (m, 5H, phenyl-H), 5.68 (s, 2H, CH_2_-C=N), 5.41 (s, 1H, OH, D_2_O exchangeable), 5.31 (s, 2H, CH_2_-OH). ^13^C NMR (125 MHz, DMSO-*d*_*6*_): δ 165.95 (C=O) 150.32 (C=N), 142.25 (triazole-C_4_), 134.42 (chlorophenyl-C_4_), 134.08 (phenyl-C_4’_), 133.48 (chlorophenyl-C_1_), 130.86 (phenyl-C_1’_), 129.78 (chlorohenyl-C_2,6_), 129.37 (phenyl-C_2’,6’_), 129.08 (chlorophenyl-C_3,5_), 128.57 (phenyl-C_3’,5’_), 126.47 (triazole-C_5_), 58.33 (CH_2_-OH), 43.33 (CH_2_-C=N). Microanalysis %: Calcd for C_18_H_15_ClN_4_O_3_ (370.79): C, 58.31; H, 4.08; Cl, 9.56; N, 15.11. Found: C, 58.59%; H, 4.24%; N, 15.23%.

#### 2-(((1-(4-chlorophenyl)-2-(4-(hydroxymethyl)-1H-1,2,3-triazol-1-yl) ethylidene)amino)-oxy)acetamide (*16*)

A solution of **8** (0.35 g, 1 mmol) and ammonia solution (33%, 0.6ml) in ethanol (5ml) was stirred at room temperature for 48 h. The solvent was completely evaporated. The obtained solid was triturated with acidified water, filtered, washed with water, dried, and crystallized from ethanol.

White solid, yield: 40%, m.p.: 144–146 °C. IR (KBr, ν-cm^-1^): 3439 (OH), 3382&3149 (NH_2_), 3149, 2981, 2932 (C-H), 1744&1676 (H_2_N-C=O), 1650 (C=N), 1595 (C=C), 1493 (N = N), 837 (C=C–Cl). ^1^H NMR (500 MHz, DMSO-*d*_*6*_): δ 8.01 (s, 1H, triazole-CH), 7.66 (d, *J* = 8.2 Hz, 2H, chlorophenyl-C_2,6_-H), 7.44 (d, *J* = 8.5 Hz, 2H, chlorophenyl-C_3,5_-H), 7.28 (s, 2H, NH_2,_ D_2_O- exchangeable), 5.72 (s, 2H, CH_2_-C=N), 5.14 (s, 1H, OH, D_2_O- exchangeable), 4.79 (s, 2H, CH_2_-C=O), 4.41 (s, 2H, CH_2_-OH). ^13^C NMR (125 MHz, DMSO-*d*_6_): δ 171.33 (C=O), 152.33 (C=N), 148.81 (triazole-C_4_), 135.36 (chlorohenyl-C_4_), 131.95 (chlorophenyl-C_1_), 129.28 (chlorohenyl-C_3,5_), 128.88 (chlorophenyl-C_2,6_), 123.95 (triazole-C_5_), 71.56 (O-CH_2_-C=O) 55.44 (CH_2_-OH), 43.56 (CH_2_- C=N). Microanalysis %: Calcd for C_13_H_14_ClN_5_O_3_ (323.74): C, 48.23; H, 4.36; Cl, 10.95; N, 21.63. Found: C, 48.45%; H, 4.49%; N, 21.90%.

#### (E)-2-(((1-(4-chlorophenyl)-2-(4-(hydroxymethyl)-1H-1,2,3-triazol-1-yl)ethylidene) amino)oxy)-N-hydroxyacetamide (*17*)

To a mixture of **8** (0.35 g, 1 mmol), and anhydrous potassium hydroxide (0.11 g, 2 mmol) in ethanol (5 ml), hydroxylamine hydrochloride (0.13 g, 2 mmol) was added and stirred at room temperature for 24 h. The solvent was completely evaporated. The obtained solid was triturated with acidified water, filtered, washed with water, dried, and crystallized from ethanol.

White solid, yield: 35%, m.p.: 120–122 °C. IR (KBr, ν-cm^-1^): 3469 (2 OH groups ), 2935, 2721 (C-H), 1626 (C=O), 1592 (C=N), 1494 (C=C), 1407 (N = N), 841 (C=C–Cl). ^1^H NMR (500 MHz, DMSO-*d*_*6*_): δ 10.75 (s, 1H, OH, D_2_O- exchangeable), 8.01 (s, 1H, triazole-CH), 7.64 (d, *J* = 8.3 Hz, 2H, chlorophenyl-C_2,6_-H), 7.43 (d, *J* = 8.1 Hz, 2H, chlorophenyl-C_3,5_-H), 7.30 (s, 1H, NH, D_2_O- exchangeable), 5.75 (s, 2H, CH_2_-C=N), 5.16 (s, 1H, OH, D_2_O- exchangeable), 4.64 (s, 2H, CH_2_-C=O), 4.40 (s, 2H, CH_2_-OH). Microanalysis %: Calcd for C_13_H_14_ClN_5_O_4_ (339.74): C, 45.96; H, 4.15; Cl, 10.43; N, 20.61. Found: C, 46.08%; H, 4.26%; N, 20.78%.

#### 2-(((1-(4-chlorophenyl)-2-(4-(hydroxymethyl)-1H-1,2,3-triazol-1-yl) ethylidene) amino) oxy) acetohydrazide (*18*)

A solution of **8** (0.35 g, 1 mmol) and hydrazine hydrate (99%, 0.3 ml, 6 mmol) in ethanol (5 ml) was stirred at room temperature for 3 h. The solvent was completely evaporated. The obtained solid was triturated with acidified water, filtered, washed with water, dried, and crystallized from ethanol.

White solid, yield: 35%, m.p.: 94–95 °C. IR (KBr, ν-cm^-1^): 3419 (OH), 3130 & 3066 (NH_2_), 2964, 2941, 2874 (C-H), 1688, 1639 (C=O), 1589 (C=N), 1557 (C=C), 1491 (N = N), 846 (C=C–Cl). ^1^H NMR (500 MHz, DMSO-*d*_*6*_): δ 9.22 (s, 1H, NH_,_ D_2_O- exchangeable), 8.01 (s, 1H, triazole-CH), 7.65 (d, *J* = 8.5 Hz, 2H, chlorophenyl-C_2,6_-H), 7.44 (d, *J* = 8.2 Hz, 2H, chlorophenyl-C_3,5_-H), 5.77 (s, 2H, CH_2_-C=N), 5.13 (s, 1H, OH, D_2_O- exchangeable), 4.68 (s, 2H, CH_2_-C=O), 4.51–4.28 (m, 4H, CH_2_-OH, NH_2,_ D_2_O- exchangeable). ^13^C NMR (125 MHz, DMSO-*d*_6_): δ 158.33 (C=O), 156.21 (C=N), 148.66 (triazole-C_4_), 136.33 (chlorophenyl-C_4_), 133.94 (chlorophenyl-C_1_), 129.96, 129.28, 126.88 (chlorophenyl-C_2,3,5,6_), 123.93 (triazole-C_5_), 71.26 (O-CH_2_-C=O), 55.35 (CH_2_-OH), 45.50 (CH_2_-C=N). Microanalysis %: Calcd for C_13_H_15_ClN_6_O_3_ (338.75): C, 46.09; H, 4.46; Cl, 10.46; N, 24.81. Found: C, 46.31%; H, 4.23%; N, 24.89%.

#### N'-(4-Substituted benzylidene)-2-(((-1-(4-chlorophenyl)-2-(4-(hydroxymethyl)-1H-1,2,3-triazol-1-yl) ethylidene) amino)oxy)acetohydrazide (19a-c)

A solution of **18** (0.34 g,1 mmol) and the appropriate benzaldehyde (1 mmol) in ethanol (5 ml) was heated under reflux for 3 h, then left to cool. The obtained crystals were filtered, washed with water, dried, and recrystallized from ethanol giving white crystals.

##### N'-(benzylidene)-2-(((-1-(4-chlorophenyl)-2-(4-(hydroxymethyl)-1H-1,2,3-triazol-1-yl) ethylidene)amino)oxy) acetohydrazide (19a)

 White solid, yield: 70%, m.p.: 222–224 °C. IR (KBr, ν-cm^-1^): 3409 (OH), 3142 (NH), 3085, 2947, 2832 (C-H), 1705 (C=O), 1610 (C=N), 1597 (C=C), 1494 (N = N), 839 (C=C–Cl). ^1^H NMR (500 MHz, DMSO-*d*_*6*_): δ 8. 68 (s, 1H, N = CH), 7.95 (t, *J* = 7.6 Hz, 2H, phenyl-C_3’,5’_-H), 7.89–7.83 (m, 3H, chlorophenyl-C_2,6_-H, triazole-CH ), 7.58–7.53 (m, 3H, phenyl-C_2’,4’,6’_-H), 7.50 (d, *J* = 8.0 Hz, 2H, chlorophenyl-C_3,5_-H), 6.00 (s, 2H, CH_2_-C=N), 5.09 (t, *J* = 5.6 Hz, 1H, OH, D_2_O- exchangeable), 4.37 (d, *J* = 5.6 Hz, 2H, CH_2_-OH), 3.51 (s, 2H, CH_2_-C=O). Microanalysis %: Calcd C_20_H_19_ClN_6_O_3_ (426.86): C, 56.28; H, 4.49; Cl, 8.30; N, 19.69. Found: C, 56.43%; H, 4.28%; N, 19.75%.

##### N'-(4-chlorobenzylidene)-2-(((-1-(4-chlorophenyl)-2-(4-(hydroxymethyl)-1H-1,2,3-triazol-1-yl) ethylidene) amino) oxy) acetohydrazide (19b)

White solid, yield: 80%, m.p.: 237–239 °C. IR (KBr, ν-cm^-1^): 3415 (OH), 3144 (NH), 3102, 2947, 2840 (C-H), 1706 (C=O), 1611 (C=N), 1597 (C=C), 1491 (N = N), 827 (C=C–Cl). ^1^H NMR (500 MHz, DMSO-*d*_*6*_): δ 11.63, 11.43 (2s, 1H, NH, D_2_O- exchangeable, E & Z isomers), 8.38, 8.19 (s, 1H, N = CH, E & Z isomers), 8.10, 7.94 (2s, 1H, triazole-CH, E & Z isomers), 7.71–7.62 (m, 4H, chlorophenyl-C_2’,6’_-H, chlorophenyl-C_2,6_-H), 7.43–7.33 (m, 4H, chlorophenyl-_3’,5’_-H, chlorophenyl-C_3,5_-H), 5.77, 5.73 (2s, 2H, CH_2_-C=N, E & Z isomers), 5.30, 4.85 (2s, 2H, CH_2_-C=O, E & Z isomers), 5.13, 5.07 (2t, *J* = 5.8, 5.9 Hz, 1H, OH, D_2_O- exchangeable, E & Z isomers), 4.48, 4.44 (2d, *J* = 5.7, 5.7 Hz, 2H, CH_2_-OH, E & Z isomers). ^13^C NMR (125 MHz, DMSO-*d*_6_): δ 170.37, 165.54 (C=O), 152.85, 151.95 (C=N), 148.65,147.03 (NH-N = CH), 143.23 (triazole-C_4_), 135.28,134.90 (chlorophenyl-C_4_), 133.14 (chlorophenyl-C_1_), 131.07 (chlorophenyl-C_1’_), 129.77, 129.66, 129.27, 128.91, 128.82 (chlorophenyl-C_2,2’,3,3’,5,5’,6,6’_), 124.06,123.87 (triazole-C_5_), 73.02,72.03 (O-CH_2_-C=O), 55.41 (CH_2_-OH), 45.03,43.55 (CH_2_-C=N). Microanalysis %: Calcd C_20_H_18_Cl_2_N_6_O_3_ (461.30): C, 52.07; H, 3.93; Cl, 15.37; N, 18.22; O, 10.40. Found: C, 52.29%; H, 3.84%; N, 18.43%.

##### N'-(4-fluorobenzylidene)-2-(((-1-(4-chlorophenyl)-2-(4-(hydroxymethyl)-1H-1,2,3-triazol-1-yl) ethylidene) amino) oxy)acetohydrazide (19c)

White solid, yield: 80%, m.p.: 228–230 °C. IR (KBr, ν-cm^-1^): 3415 (OH), 3144 (NH), 3102, 2947, 2840 (C-H), 1706 (C=O), 1611 (C=N), 1597 (C=C), 1491 (N = N), 827 (C=C=C–Cl). ^1^H NMR (500 MHz, DMSO-*d*_*6*_): δ 11.63, 11.43 (2s, 1H, NH, D_2_O- exchangeable, E & Z isomers), 8.38, 8.19 (s, 1H, N = CH, E & Z isomers), 8.10, 7.94 (2s, 1H, triazole-CH, E & Z isomers), 7.71–7.62 (m, 4H, chlorophenyl-C_2’,6’_-H, chlorophenyl-C_2,6_-H), 7.43–7.33 (m, 4H, chlorophenyl-_3’,5’_-H, chlorophenyl-C_3,5_-H), 5.77, 5.73 (2s, 2H, CH_2_-C=N, E & Z isomers), 5.30, 4.85 (2s, 2H, CH_2_-C=O, E & Z isomers), 5.13, 5.07 (2t, *J* = 5.8, 5.9 Hz, 1H, OH, D_2_O- exchangeable, E & Z isomers), 4.48, 4.44 (2d, *J* = 5.7, 5.7 Hz, 2H, CH_2_-OH, E & Z isomers). ^13^C NMR (125 MHz, DMSO-*d*_6_): δ170.36, 165.39 (C=O), 164.55, 162.59 (J = 245.

Hz, fluorophenyl-C_4_’), 152.86, 151.91 (CH_2_-C=N), 148.85, 147.34 (NH-N = CH), 143.35(triazole-C_4_), 135.29, 134.49 (chlorophenyl-C_4_), 132.04, 131.78 (fluorophenyl-C_1’_), 131.10(chlorophenyl-C_1_), 129.91, 129.84, 129.67, 129.60 (J = 8.7, 8.7 Hz, fluorophenyl-C_2’,6’_),129.33,129.28, 128.89, 128.80 (chlorophenyl-C_2,3,5,6_), 124.26, 124.04 (triazole-C_5_), 116.59,116.50, 116.42, 116.33 (J = 21.5, 21.5 Hz, fluorophenyl-C_3’,5’_), 73.08, 72.04 (O-CH_2_-C=O),55.43 (CH_2_-OH), 43.55 (CH_2_-C=N). Microanalysis %: Calcd C_20_H_18_ClFN_6_O_3_ (444.85): C, 54.00; H, 4.08; Cl, 7.97; F, 4.27; N, 18.89; O, 10.79. Found: C, 53.89%; H, 4.23%; N, 19.07%.

### Biological evaluation

#### In vitro antifungal activity

The minimal inhibitory concentration (MIC) of the target compounds was estimated using the serial microdilution technique in a 96-flat-bottom well microdilution plate in accordance with EUCAST microdilution procedures for antifungal testing of *C.albicans*^79^. The yeasts were cultured for 48 h at 35 °C. The fungal suspension was prepared in double strength RPMI (gibco, UK) 1640 medium with a count of 10^5^ CFU/ml. The compounds of interest were dissolved in DMSO before being serially diluted in a growth medium. The target compounds were twofold serially diluted from left to right (according to the series on the plate) in decreasing concentration). Each well received 100µl fungal suspension in double strength RPMI 1640 medium and 100µl double concentrated compounds (to allow for a 50% [1:1] dilution). Each row represents one selected isolate experiment. The first well in each row was a positive control containing sterile distilled water instead of target compounds to control the adequacy of the broth to support the growth of the organism. The last well was a negative control to check the sterility of the procedure containing 100µl double strength RPMI 1640 medium and 100µl sterile distilled water. Each compound was tested in duplicated manner. Following incubation at 35 °C for 24h, the optical density (OD 600 nm) in each well was assessed using a spectrophotometer. The MICs were established as the lowest drug concentrations required to suppress fungal cell growth by 50% as compared to a drug-free control^80^.

The cut-off for 50% growth inhibition is calculated as follows using the mean OD value of the negative controls (mean OD-NC) and mean of positive growth wells (mean OD-PG) controls as follows:

$${\text{50\% }}\,{\mathrm{OD}}\,{\mathrm{endpoint}}\,{\mathrm{determination:}}\,{\mathrm{((mean}}\,{\text{OD - PG)}}$$

#### Ergosterol inhibition assay

The entire intracellular sterols were extracted using the technique outlined by Breivik and Owades^73^, with slight adjustments. In brief, a single *C. albicans* colony from an overnight Sabouraud dextrose agar plate culture was used to inoculate 50 ml of Sabouraud dextrose broth (Himedia®) containing (1*MIC & 0.5*MIC) of compound 11 and reference drug fluconazole. The colonies were incubated at 35 °C for 16 h with shaking. The stationary-phase cells were collected by centrifugation at 2,700 rpm for 5 min (Cooling centrifuge Sigma® 2-16KL, Germany) and washed once with sterile distilled water. The total weight of the cell pellets was determined. 3 ml of 25% alcoholic potassium hydroxide solution (prepared by 25 g KOH and 36 ml pure distilled water, raised to a volume of 100 ml with absolute ethanol) was added to each container and vortexed for a minute. The cell suspensions were afterwards incubated at 85 °C for 1 h. Tubes were permitted to cool down to room temperature after incubation. One milliliter of pure deionized water and three milliliters of n-heptane were added together with the aim of extracting the sterols, which was then thoroughly mixed for three minutes using vortex device. After that, the n-heptane layer was moved to a clear borosilicate glass screw-cap tube and stored at -20 °C for up to 24 h. A 1 ml aliquot of sterol extract was diluted with fivefold in 100% ethanol before being scanned spectrophotometrically (triplicate measuring) with a NanoDrop device (NanoDrop C, Thermo Fisher Scientific®, USA) between 200 and 300 nm (nano drop). The experiment was done in duplicate. Ergosterol content was calculated based on the presence of ergosterol and the late sterol intermediate 24(28) Dehydroergosterol (DHE) in the isolated sample. There was a dose-dependent decline in the concentration of ergosterol, which correlated to lower ergosterol content^50,73^.

The following equations were used to assess ergosterol concentration as a percentage of the cells wet weight:

$$\% {\text{ ergosterol }} + \, \% { 24}\left( {{28}} \right){\text{ DHE }} = \, \left[ {\left( {A_{{{281}.{5}}} /{29}0} \right) \, \times F} \right]/{\text{pellet weight}},$$

$$\% { 24}\left( {{28}} \right){\text{ DHE }} = \, \left[ {\left( {A_{{{23}0}} /{518}} \right) \, \times F} \right]/{\text{pellet weight}},$$

$$\% {\text{ ergosterol }} = \, \left[ {\% {\text{ergosterol }} + \, \% { 24}\left( {{28}} \right){\text{ DHE}}} \right] \, - \, \% { 24}\left( {{28}} \right){\text{ DHE}},$$

where *F* is the factor for dilution in ethanol and 290 and 518 are the E values (in percent per centimeter) determined for crystalline ergosterol and 24(28) DHE, respectively.

#### Antifungal drug combination

Checkerboard titration method using 96-well polypropylene microtiter plates were used to study the activity of fluconazole combination with compound 11. The concentrations used ranged from four times the MIC (determined by the broth microdilution technique) to at least 1/8 times the MIC of each antibiotic corresponding to the isolate under testing. The stock solutions of fluconazole and compound **11** were two-fold serially diluted with Roswell Park Memorial Institute Medium, RPMI. Stock concentrations prepared four times the required final concentration ranges to compensate for the subsequent dilutions. Inoculum were prepared by diluting an overnight broth culture of each of the selected isolates in double strength RPMI to reach a final inoculum of 10^5^ CFU/ml. Fluconazole was distributed in decreasing concentration vertically, while compound **11** was distributed in decreasing concentration horizontally. One microtiter plate was used for each isolate. Each well received 50 μl of 4 × needed fluconazole concentration, 50 μl of 4 × needed compound **11** concentration, and 100 μl of the inoculated isolate in double strength RPMI. A1 well was a positive control containing sterile distilled water instead of the combined compounds to control the adequacy of the broth to support the growth of the organism while H12 well was a negative control to check the sterility of the procedure containing 100µl double strength RPMI and 100µl sterile distilled water. The system was duplicated and then incubated at 35 ºC for 48 h. Then the plate was examined for growth using automated microplate reader at 600 nm wavelength^55,56^. The fraction inhibitory concentration index (FICI) for combinations was calculated according to the equation:

$$\Sigma {\text{FICI }} = {\text{ FIC A }} + {\text{ FIC B }} = {\text{ A }}/{\text{ MICA }} + {\text{ B }}/{\text{ MICB}},$$

where A and B are the MICs of drugs A and B, respectively, in the combination, MICA and MICB are the MICs of drugs A and B, respectively, alone, and FICA and FICB are the FICs of drugs A and B, respectively.

#### In vitro MTT cytotoxicity assay

The cytotoxic effect of the above-mentioned compounds was assayed using three different human cell lines; human lung Adenocarcinoma A549, colorectal adenocarcinoma Caco-2, and breast cancer MCF7. Cells were cultured and maintained in DMEM high glucose (Biowest, France) supplemented with 10% FBS (Biowest, France), 100U/ml penicillin and 1% streptomycin. All cancer cells were seeded at a density of 5 × 10^3^ cells/well in sterile 96-well flat bottom tissue culture plates the day before treatment. After 24 h, serial concentrations of the tested compounds, including docetaxel as reference drug (positive control), were added to the seeded cells and were incubated together for 72 h at 37 ºC in a 5% CO_2_ incubator. Thereafter, MTT (Biobasic, Canada) dissolved in PBS was added to each well at a final concentration of 0.5 mg/ml, the plates were then incubated at 37 ºC for 3 h in the dark. MTT solution was then removed, 100 µl DMSO was added to dissolve the formed formazan crystals and the absorbance was measured with a microplate reader (BioTek, USA)^57^. The percent viability was calculated for each concentration relative to the control DMSO treated cells. All experiments were done at least in duplicates. Docetaxel served as positive control. The IC_50_ values of each of the tested compounds were calculated using the Graphpad software.

#### Cell cycle analysis using flow cytometry

PI/RNA reagent (cell signaling) was used to do cell cycle analysis. The pellet was resuspended in 1 mL of PBS per 10^6 cells after the cells were centrifuged for five minutes at 1800 rpm to remove the supernatant. The particle was re-suspended for 60 min in 1 mL of cold ice 90% methanol following centrifugation and the removal of the supernatant. After centrifuging methanol-fixed cells for five minutes at room temperature at 1800 rpm, the cell pellet was resuspended in one PBS. The pellet was resuspended in 200 µL of cell cycle reagent and harvested at room temperature for 30 min after the cells were centrifuged once more at 1800 rpm over five minutes. Supernatant was discarded​ (30). After being moved to a 5 mL flow tube, cell suspension models were prepared for Flowcytometry analysis. The stained cells were assessed using the BD FACS flow cytometer (BD Biosciences)^81^.

#### Apoptosis analysis (Annexin V-FITC assay)

The Annexin V-FITC apoptosis recognition kit (Miltenyi Biotec) was conducted to assess apoptosis. Sorafenib, hydroxychloroquine, and the synergistic combination dosage were conducted to cure MDA-MB-231 breast tumor cells for 48 h. Following centrifugation, the cells were resuspended in binding buffer, harvested using fluorescein isothiocyanate (FITC), and classified using Annexin V for 15 min at room temperature in the dark. Following two washes with 1 × PBS, the cells were resuspended in binding buffer, propidium iodide was added, and they were harvested over 15 min at room temperature in dark (29). The marked cells were examined via BD FACS flow cytometer (BD Biosciences)^81^.

#### Identification of synergism, antagonism, and dose reduction in drug combination

Anticancer effect of the above-mentioned compound **11** was assayed alone and as adjuvant agent when combined with docetaxel against lung adenocarcinoma A549, and colorectal adenocarcinoma Caco-2. The MTT assay was conducted as described above. Cells (5 × 10^3^ /well) were seeded and incubated for 24 h, serial concentrations of the tested compound **11** alone and in combination with docetaxel were incubated with these cell lines for 72 h at 37 ºC in 5% carbon dioxide incubator. MTT was then added for 3 h as described above and the absorbance was read after dissolving the crystals with DMSO. Each experiment was done in duplicates. The dose–response information for individual medications in A549 lung and CaCo2 colon tumor cells were used to determine the drug doses used in combination tests. As demonstrated by Chou, fraction affected (Fa) quantities were estimated as cell viability percentage inhibition in comparison to the control. The Chou Tala lay Combination Index (CI) approach was used to identify if drug combinations were antagonistic, additive, or synergistic. The CompuSyn software (http://www.combosyn.com), which is rely on the next equation:

$$\mathrm{C}\mathrm{I} = {\left.\left(\frac{(\mathrm{D})1}{(\mathrm{D}\mathrm{x} )1}\right.\right)}+\left.\left(\frac{(\mathrm{D})2}{(\mathrm{D}\mathrm{x} )2}\right.\right)$$

where the concentrations of Drugs 1 and 2 in the combination to generate a Fa value of x are denoted by (D)1 and (D)2. When used as separate medicines, (Dx)1 and (Dx)2 represent the concentrations of Drug 1 and Drug 2 that generate the same effect (x). CI values reveal antagonism, additivity, and synergism, respectively.

The possibility for lowering the dosage of individual medications when taken together to attain a particular effect level in comparison to their use as single agents was measured using the Dose Reduction Index (DRI) (28). In order to measure the likelihood that the doses of each medicine in a synergistic combination might be lowered through specific consequence level in comparison to the doses of each drug solely, the dose reduction index (DRI) was formally created. Using CompuSyn software (http://www.combosyn.com), the dose-reduction index (DRI) is primarily rely on the next equation:

$$\mathrm{C}\mathrm{I} = {\left.\left(\frac{(\mathrm{D})1}{(\mathrm{D}\mathrm{x}1}\right.\right)}+\left.\left(\frac{(\mathrm{D})2}{(\mathrm{D}\mathrm{x}2}\right.\right)={\left.\left(\frac{1}{\mathrm{D}\mathrm{R}\mathrm{I}1}\right.\right)}+\left.\left(\frac{1}{\mathrm{D}\mathrm{R}\mathrm{I}2}\right.\right)$$

where D₁ and D₂ are the doses of drugs 1 and 2 taken together to achieve a specific level of effect. The doses of drug 1 and drug 2 alone resulting in the similar amount of action are Dx₁ and Dx₂. The dose-reduction indices for drugs 1 and 2 are DRI₁ and DRI₂, respectively^58^.

### Molecular docking studies

The crystallographic structure of 14α-sterol demethylase from *C.albicans* complex with VT-1161 (PDB ID: 5TZ1) was retrieved from Protein Data Bank. All calculations were performed using MOE 2020.09 software^74^. The compounds were prepared by hydrogens addition, partial charges calculation and energy minimization using Amber10: EHT Force Field with root mean square (RMSD) gradient of 0.1 kcal/mol. Besides, preparation of proteins was performed by removing the repeating chains and water molecules. For optimizing structural issues, 3D protonation and calculation of partial charges, MOE QuickPrep protocol was used. The MOE Dock protocol was used to determine the best poses and binding score values of the selected compounds using induced fit refinement. To validate the docking technique, the co-crystallized ligand VT-1161 was re-docked into the LDM active site. The triangle matcher placement technique and London dG were used as the primary scoring functions for the compound. The induced fit technique with the affinity dG scoring function was also used as an additional refinement step. Furthermore, docking poses were evaluated, and interactions with the active site were investigated. Finally, the highest scoring postures that fit into the active site and had favorable ligand-enzyme interactions were chosen.

### In silico prediction of the physicochemical properties, drug likeness score, pharmacokinetics, and toxicity profile

In the current study, the most active compounds (**5b, 8, 11,** and **19c**) were subjected to molecular properties prediction using the Molinspiration online software^74^ and drug-likeness and solubility parameter calculation using the Molsoft software^73^. Additionally, in order to filter and evaluate their total potential as a drug candidate, ADME profiling by the pre-ADMET calculator^75^ and toxicity factors such as mutagenic, tumorigenic, reproductive, and irritant properties were carried out using Osiris toxicity prediction.

## Supplementary Information

Below is the link to the electronic supplementary material.

Supplementary Material 1

## Data Availability

All data generated or analyzed during this study are included in this published article [and its supplementary information files].
