# Supplementary material for: Design, synthesis, molecular modelling and biological evaluation of oxiconazole-based 1,2,3-triazoles as dually acting antifungal and antitumor agents
Source: Sci Rep. 2026 Aug 14;16:25480. doi: 10.1038/s41598-026-65234-9 (PMC13476240; doi:10.1038/s41598-026-65234-9)
Supplement: Supplementary file 1 [file 41598_2026_65234_MOESM1_ESM.docx]

Table of Contents

[S1. Graphs for calculation of combination index	4](#_Toc222357192)

[S2. The IR,^1^H and ^13^C NMR spectra, and CHN analysis of compounds 4-19c	5](#_Toc222357193)

[^1^H-NMR of compound 4	5](#_Toc222357194)

[^13^C NMR of compound **4**	6](#_Toc222357195)

[IR of compound **4**	7](#_Toc222357196)

[^1^H-NMR of compound **5a**	8](#_Toc222357197)

[^13^C NMR of compound **5a**	9](#_Toc222357198)

[IR of compound **5a**	10](#_Toc222357199)

[^1^H-NMR of compound 5b	11](#_Toc222357200)

[^13^C NMR of compound **5b**	12](#_Toc222357201)

[IR of compound **5b**	13](#_Toc222357202)

[^1^H-NMR of compound **5c**	14](#_Toc222357203)

[^13^C NMR of compound **5c**	15](#_Toc222357204)

[IR of compound **5c**	16](#_Toc222357205)

[^1^H-NMR of compound **6**	17](#_Toc222357206)

[^13^C NMR of compound **6**	18](#_Toc222357207)

[IR of compound **6**	19](#_Toc222357208)

[^1^H-NMR of compound **7**	20](#_Toc222357209)

[^13^C NMR of compound **7**	21](#_Toc222357210)

[IR of compound **7**	22](#_Toc222357211)

[^1^H-NMR of compound **8**	23](#_Toc222357212)

[IR of compound **8**	24](#_Toc222357213)

[^1^H-NMR of compound **9**	25](#_Toc222357214)

[^13^C NMR of compound **9**	26](#_Toc222357215)

[IR of compound **9**	27](#_Toc222357216)

[^1^H-NMR of compound **10**	28](#_Toc222357217)

[^13^C NMR of compound **10**	29](#_Toc222357218)

[IR of compound **10**	30](#_Toc222357219)

[^1^H-NMR of compound **11**	31](#_Toc222357220)

[^13^C NMR of compound **11**	32](#_Toc222357221)

[IR of compound **11**	33](#_Toc222357222)

[^1^H-NMR of compound **12**	34](#_Toc222357223)

[IR of compound **12**	35](#_Toc222357224)

[^1^H-NMR of compound **13**	36](#_Toc222357225)

[^13^C NMR of compound **13**	37](#_Toc222357226)

[IR of compound **13**	38](#_Toc222357227)

[^1^H-NMR of compound **14**	39](#_Toc222357228)

[IR of compound **14**	40](#_Toc222357229)

[^1^H-NMR of compound **15**	41](#_Toc222357230)

[^13^C NMR of compound **15**	42](#_Toc222357231)

[IR of compound **15**	43](#_Toc222357232)

[^1^H-NMR of compound **16**	44](#_Toc222357233)

[^13^C NMR of compound **16**	45](#_Toc222357234)

[IR of compound **16**	46](#_Toc222357235)

[1H-NMR of compound 17	47](#_Toc222357236)

[IR of compound 17	48](#_Toc222357237)

[^1^H-NMR of compound **18**	49](#_Toc222357238)

[^13^C NMR of compound 18	50](#_Toc222357239)

[IR of compound 18	51](#_Toc222357240)

[^1^H-NMR of compound **19a**	52](#_Toc222357241)

[IR of compound **19a**	53](#_Toc222357242)

[^1^H-NMR of compound **19b**	54](#_Toc222357243)

[^13^C NMR of compound **19b**	55](#_Toc222357244)

[IR of compound **19b**	56](#_Toc222357245)

[^1^H-NMR of compound **19c**	57](#_Toc222357246)

[^13^C NMR of compound **19c**	58](#_Toc222357247)

[IR of compound **19c**	59](#_Toc222357248)

[CHN analysis of the synthesized compounds **4 - 19c**	60](#_Toc222357249)

[References	61](#_Toc222357250)

# S1. Graphs for calculation of combination index

Dose-Effect Curve

Median-Effect Plot

Combination Index Plot

Dose-Effect Curve

Median-Effect Plot

Combination Index Plot

**Figure S1**. Effect of compound **11** on IC_50_ of docetaxel against (A) A549, (B)Caco-2.

# S2. The IR,^1^H and ^13^C NMR spectra, and CHN analysis of compounds 4-19c

# ^1^H-NMR of compound 4

# ^13^C NMR of compound **4**

# IR of compound **4**

# ^1^H-NMR of compound **5a**

# ^13^C NMR of compound **5a**

# IR of compound **5a**

^1^H-NMR of compound 5b

# ^13^C NMR of compound **5b**

# IR of compound **5b**

# ^1^H-NMR of compound **5c**

# ^13^C NMR of compound **5c**

# IR of compound **5c**

# ^1^H-NMR of compound **6**

# ^13^C NMR of compound **6**

# IR of compound **6**

# ^1^H-NMR of compound **7**

# ^13^C NMR of compound **7**

# IR of compound **7**

# ^1^H-NMR of compound **8**

# IR of compound **8**

# ^1^H-NMR of compound **9**

# ^13^C NMR of compound **9**

# IR of compound **9**

# ^1^H-NMR of compound **10**

# ^13^C NMR of compound **10**

# IR of compound **10**

# ^1^H-NMR of compound **11**

# ^13^C NMR of compound **11**

# IR of compound **11**

# ^1^H-NMR of compound **12**

# IR of compound **12**

# ^1^H-NMR of compound **13**

# ^13^C NMR of compound **13**

# IR of compound **13**

# ^1^H-NMR of compound **14**

# IR of compound **14**

# ^1^H-NMR of compound **15**

# ^13^C NMR of compound **15**

# IR of compound **15**

# ^1^H-NMR of compound **16**

# ^13^C NMR of compound **16**

# IR of compound **16**

1H-NMR of compound 17

IR of compound 17

# ^1^H-NMR of compound **18**

^13^C NMR of compound 18

IR of compound 18

# ^1^H-NMR of compound **19a**

# IR of compound **19a**

# ^1^H-NMR of compound **19b**

# ^13^C NMR of compound **19b**

# IR of compound **19b**

# ^1^H-NMR of compound **19c**

# ^13^C NMR of compound **19c**

# IR of compound **19c**

# CHN analysis of the synthesized compounds **4 - 19c**

| **`** | **C%** | **H%** | **N%** |
| --- | --- | --- | --- |
| 4 | 52.68 | 4.27 | 16.93 |
| 5a | 59.86 | 4.97 | 20.71 |
| 5b | 57.02 | 4.31 | 19.70 |
| 5c | 54.61 | 4.25 | 18.75 |
| 6 | 49.80 | 4.29 | 21.28 |
| 7 | 44.60 | 4.20 | 25.72 |
| 8 | 51.35 | 4.98 | 16.04 |
| 9 | 49.85 | 4.70 | 16.73 |
| 10 | 60.42 | 4.96 | 15.94 |
| 11 | 55.43 | 4.25 | 14.59 |
| 12 | 55.40 | 4.41 | 18.64 |
| 13 | 40.47 | 3.29 | 14.67 |
| 14 | 45.73 | 3.68 | 16.50 |
| 15 | 58.59 | 4.24 | 15.23 |
| 16 | 48.45 | 4.49 | 21.90 |
| 17 | 46.08 | 4.26 | 20.78 |
| 18 | 46.31 | 4.63 | 24.89 |
| 19a | 56.43 | 4.28 | 19.75 |
| 19b | 52.29 | 3.84 | 18.43 |
| 19c | 53.89 | 4.23 | 19.07 |

# References

[1]	H. van de Waterbeemd and E. Gifford, "ADMET in silico modelling: towards prediction paradise?," *Nat Rev Drug Discov,* vol. 2, no. 3, pp. 192-204, Mar 2003.

[2]	L. L. G. Ferreira and A. D. Andricopulo, "ADMET modeling approaches in drug discovery," *Drug Discov Today,* vol. 24, no. 5, pp. 1157-1165, May 2019.

[3]	C. A. Lipinski, F. Lombardo, B. W. Dominy, and P. J. Feeney, "Experimental and computational approaches to estimate solubility and permeability in drug discovery and development settings," *Adv Drug Deliv Rev,* vol. 46, no. 1-3, pp. 3-26, Mar 1 2001.

[4]	S. Tian, J. Wang, Y. Li, D. Li, L. Xu, and T. Hou, "The application of in silico drug-likeness predictions in pharmaceutical research," *Adv Drug Deliv Rev,* vol. 86, pp. 2-10, Jun 23 2015.

[5]	D. F. Veber, S. R. Johnson, H. Y. Cheng, B. R. Smith, K. W. Ward, and K. D. Kopple, "Molecular properties that influence the oral bioavailability of drug candidates," *J Med Chem,* vol. 45, no. 12, pp. 2615-23, Jun 6 2002.

[6]	D. E. Clark and S. D. Pickett, "Computational methods for the prediction of 'drug-likeness'," *Drug Discov Today,* vol. 5, no. 2, pp. 49-58, Feb 2000.

[7]	T. Hou, J. Wang, W. Zhang, and X. Xu, "ADME evaluation in drug discovery. 7. Prediction of oral absorption by correlation and classification," *J Chem Inf Model,* vol. 47, no. 1, pp. 208-18, Jan-Feb 2007.

[8]	S. Stegemann, F. Leveiller, D. Franchi, H. de Jong, and H. Linden, "When poor solubility becomes an issue: from early stage to proof of concept," *Eur J Pharm Sci,* vol. 31, no. 5, pp. 249-61, Aug 2007.

[9]	O. N. Breivik and J. L. Owades, "Yeast Analysis, Spectrophotometric Semimicrodetermination of Ergosterol in Yeast," *Journal of Agricultural and Food Chemistry,* vol. 5, no. 5, pp. 360-363, 1957/05/01 2002.

[10]	q. chemical computing group inc.: Montreal, canada., "Molecular operating environment (MOE), 2020.09, chemical computing group inc.: Montreal, qc, canada. <http://www.Chemcomp.Com>," 2020.09 ed, 2020.

[11]	J. L. Rodríguez-Tudela *et al.*, "Method for the determination of minimum inhibitory concentration (MIC) by broth dilution of fermentative yeasts," *Clinical Microbiology and Infection,* vol. 9, no. 8, pp. i-viii, 2003.

[12]	T. Sander, J. Freyss, M. von Korff, and C. Rufener, "DataWarrior: an open-source program for chemistry aware data visualization and analysis," *J Chem Inf Model,* vol. 55, no. 2, pp. 460-73, Feb 23 2015.
